# The Role of the Transmembrane RING Finger Proteins in Cellular and Organelle Function

**DOI:** 10.3390/membranes1040354

**Published:** 2011-12-09

**Authors:** Nobuhiro Nakamura

**Affiliations:** Department of Biological Sciences, Tokyo Institute of Technology, 4259-B13 Nagatsuta-cho, Midori-ku, Yokohama 226-8501, Japan; E-Mail: nnakamur@bio.titech.ac.jp; Tel.: +81-45-924-5726; Fax: +81-45-924-5824

**Keywords:** endocytosis, ERAD, immune regulation, membrane trafficking, mitochondrial dynamics, proteasome, quality control, RNF, ubiquitin, ubiquitin ligase

## Abstract

A large number of RING finger (RNF) proteins are present in eukaryotic cells and the majority of them are believed to act as E3 ubiquitin ligases. In humans, 49 RNF proteins are predicted to contain transmembrane domains, several of which are specifically localized to membrane compartments in the secretory and endocytic pathways, as well as to mitochondria and peroxisomes. They are thought to be molecular regulators of the organization and integrity of the functions and dynamic architecture of cellular membrane and membranous organelles. Emerging evidence has suggested that transmembrane RNF proteins control the stability, trafficking and activity of proteins that are involved in many aspects of cellular and physiological processes. This review summarizes the current knowledge of mammalian transmembrane RNF proteins, focusing on their roles and significance.

## 1. Introduction

**Figure 1:**
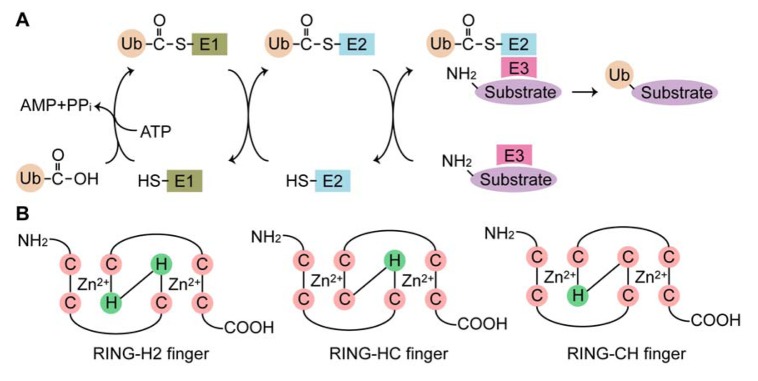
Schematic representations of the ubiquitination reaction and the RNF domain structures. (**A**) The biochemical steps in ubiquitination are illustrated. Ub is activated at its C-terminal glycine residue (Gly76) in an ATP-dependent manner by E1. In an ATP-hydrolyzing reaction, a Ub adenylate intermediate is formed, followed by the binding of Ub to a specific cysteine residue of E1. Ub is then transferred to an active site cysteine residue of E2, preserving the high energy thioester bond. The substrate is recognized by E3, which also recruits the E2–Ub complex. Finally, Ub is linked by its C-terminus in an isopeptide linkage to an ε-amino group of lysine residues on the substrate protein. (**B**) A simple comparison of the cross-brace arrangements of the RING-H2, RING-HC and RING-CH finger motifs. C and H indicate the conserved Zn^2+^-coordinating cysteine and histidine residues.

Ubiquitination is a posttranslational modification that mediates the covalent attachment of ubiquitin (Ub), a small, highly conserved, cytoplasmic protein of 76 amino acid residues, to target proteins. This conjugation is catalyzed by the sequential action of three enzymes: Ub-activating (E1) enzyme, Ub-conjugating (E2) enzyme and Ub ligase (E3). In the initial step, Ub is activated in an ATP-dependent manner by the E1 enzyme, leading to the formation of a high energy thioester bond between the C-terminal glycine (Gly76) of Ub and the cysteine residue in the active site of the E1 enzyme. The activated Ub is transferred to the catalytic cysteine residues of the E2 enzyme. The Ub moiety is then covalently attached to a target protein, with assistance of the E3 enzyme, by an isopeptide bond between the C-terminus of Ub and an ε-amino group of a lysine residue of the target protein (Figure 1(A)). Multiple rounds of the ubiquitination reaction lengthen the Ub chain (polyubiquitination) [1]. Occasionally, the extension of the Ub chain is catalyzed by Ub chain assembly factors (E4) [2]. Ub contains seven lysine residues (Lys6, Lys11, Lys27, Lys29, Lys33, Lys48 and Lys63), any of which can be used to form poly-Ub chains [3]. However, only the Lys48 and Lys63 linkages have been elucidated to date. The Lys48-linked poly-Ub chain serves mainly as a signal that allows the ubiquitinated protein to be degraded by the 26S proteasome [4,5], whereas the Lys63-linked chain has non-proteolytic functions, such as DNA repair, signal transduction and endocytosis [6,7,8]. (An) The attachment of a single Ub molecule (monoubiquitination) plays a regulatory role in protein trafficking and transcription [9]. Ubiquitination can be reversed by the deubiquitinating enzymes, a group of proteases that cleave isopepetide bonds between Ub and a target protein or within a poly-Ub chain [10]. In addition to Ub, certain E3 enzymes catalyze conjugations of Ub-like modifiers, such as small Ub-like modifier (SUMO), neural precursor cell-expressed developmentally downregulated protein 8 (Nedd8) and interferon-stimulated gene 15 kDa (ISG-15), in a manner similar to ubiquitination. These modifications result in distinct functions in a number of different cellular processes [11].

The E3 enzymes provide substrate specificity during ubiquitination, which is one reason for the presence of such a large number of the E3 enzymes compared to the E1 and E2 enzymes. Humans contain only one form of the E1 enzyme, at least 30 E2 enzymes and more than 350 E3 enzymes. The E3 enzymes are grouped into three families based on the presence of the E3 catalytic core domain: the homology to E6AP carboxyl terminus (HECT), the really interesting novel gene (RING) finger (RNF) and the U-box protein families [12]. The HECT domain is comprised of approximately 350 amino acid residues, with an E2-binding site and an active cysteine residue. Among the E3 enzymes, the HECT proteins are unique in that they form a Ub thioester intermediate via their active cysteine residues prior to transferring Ub to the substrate proteins [13]. The RNF domain is comprised of 40–80 amino acid residues with eight conserved cysteine and histidine residues that coordinate two zinc ions to form a unique three-dimensional structure known as a “cross-brace” [14,15,16,17]. The RNF domain is classified into at least three subgroups based on the presence of cysteine and histidine residues in the fourth and fifth positions: C_3_HC_4_ (RING-HC), C_3_H_2_C_3_ (RING-H2) and C_4_HC_3_ (RING-CH or RINGv) fingers (Figure 1(B)). The RNF domain serves as a scaffold for binding to E2 enzymes in close proximity to substrate proteins, which enables efficient transfer of Ub to the substrates. The U-box domain structurally resembles the RNF domain, but lacks the zinc-ion-chelating cysteine and histidine residues [18]. The human genome encodes approximately 300 RNF proteins, many of which are soluble proteins with a variety of cellular functions, including oncogenesis, development, signal transduction, the cell cycle and apoptosis. According to recent reports [19,20,21] and data base mining, at least 49 RNF proteins have hydrophobic regions predicted to be transmembrane domains, implying that they are embedded in the cellular membrane and directly participate in the biological processes of both the cellular membrane and membranous organelles. This review summarizes current knowledge of the transmembrane RNF proteins in mammals and discusses their significance in terms of organelle function and morphology.

## 2. Classification and Domain Structures of the Transmembrane RNF Proteins

**Figure 2:**
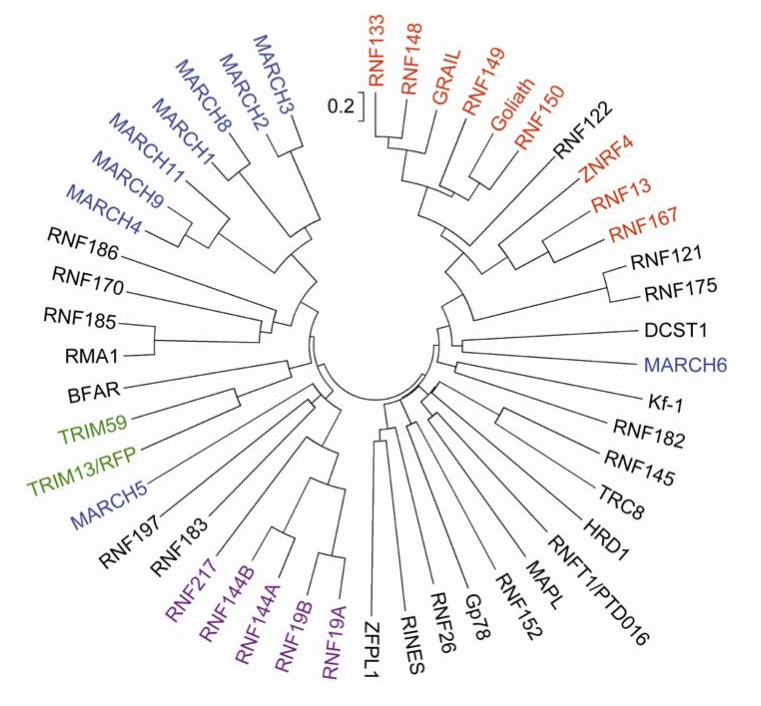
Phylogenetic analysis of putative human transmembrane RNF proteins. The tree was constructed by the neighbor-joining method with ClustalW [22] and MEGA4 [23] using 5,000 bootstrap resamplings. The scale bar indicates 0.2 amino acid substitutions per each amino acid position. Members of the TRIM, PA-TM-RING, MARCH and RBR families are indicated in green, red, blue and purple fonts, respectively.

A phylogenetic tree analysis indicated that more than half of the human transmembrane RNF proteins are grouped into a small number of structurally related clans, which include members of the tripartite motif-containing (TRIM), PA-TM-RING, RING between RNFs (RBR) and membrane-associated RING-CH (MARCH) families (Figure 2). Moderate sequence homology is observed between RMA1/RNF5 and RNF185 (∼60%) and between RNF121 and RNF175 (∼71%). The rest share little sequence homology, with the exception of the RNF domain, suggesting their diverse subcellular localization and functions.

### 2.1. The TRIM Family Members

**Figure 3:**
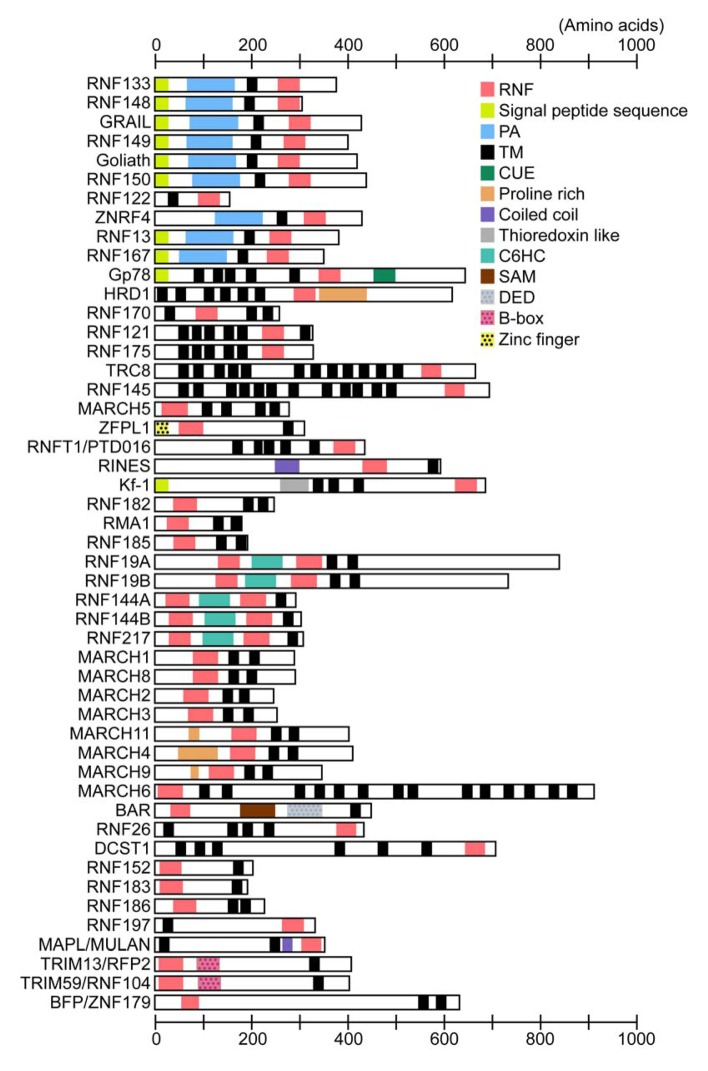
Comparison of the domain structures of putative human transmembrane RNF proteins. Information on the domain structure of each RNF protein was obtained from the ENSEMBL (http://www.ensembl.org [28]) and NCBI (http://www.ncbi.nlm.nih.gov [29]) databases. Abbreviations: PA, protease-associated domain; TM, putative transmembrane domain; CUE, coupling of Ub conjugation to ER degradation; SAM, sterile alpha motif; DED, death effecter domain.

TRIM proteins [also termed RNF, B box and coiled-coil (RBCC) proteins] are comprised of a group of proteins containing three characteristic structures, the RING-HC finger domain followed by one or two B-box domains and a coiled-coil region (Figure 3). There are more than 60 human TRIM proteins, several of which can act as E3 enzymes [24]. TRIM proteins have a single type 2 B-box (B-box2) or tandem type 1 and type 2 B-boxes. The B-boxes are zinc-finger-like motifs with the consensus sequences C5(C/H)H2 (type 1) and CHC(D/C)C2H2 (type 2), which bind two zinc ions in a cross-brace manner similar to the RING domain. The structural similarity suggests that the B-boxes and RNF domains have evolved from a common ancestor [25]. Although no clear function of the B-box domain has as yet been identified, its positioning close to the RNF domain suggests that the B-box domain may modify E3 activity. The coiled-coil regions are important for the subcellular localization and oligomerization of TRIM proteins [26]. TRIM proteins are grouped into two classes based on the structures of their C-terminal extensions: one group has an SPRY domain of unknown function and the other group has a variety of domain structures [27]. The Ret finger protein (RFP)2/TRIM13/Leu5/RFN77 and TRIM59/RNF104 belong to the latter group, and do not have any obvious domain structure apart from the hydrophobic regions (Figure 3).

### 2.2. The PA-TM-RING Family Members

The PA-TM-RING family is defined by three conserved domains, the protease-associated (PA) domain, the transmembrane domain and the RING-H2 finger domain (Figure 3) [30,31]. The PA-TM-RING family is comprised of 9 proteins [Goliath/RNF130, gene related to anergy in lymphocytes (GRAIL)/RNF128, RNF133, RNF148, RNF149, RNF150, RNF167, RNF13 and zinc and ring finger 4 (ZNRF4)/Nixin/RNF204], and most of them possess apparent signal peptide sequences at their N-termini (Figure 3). RNF122 is an anomalistic member of the PA-TM-RING family, in that it lacks both the signal peptide sequence and PA domain. PA-TM-RING proteins are unique in containing an extracellular or luminal domain (Figure 3). The extracellular or luminal PA domain is also found in several receptors and peptidases in yeast, metazoans and plants [32]. The PA domain is proposed to serve as a protein–protein interaction module. Indeed, the PA domain of GRAIL facilitates the recognition of and binding to its substrate proteins (*i.e.*, CD154/CD40L and CD83) [33,34]. RNF13 has a nuclear localization signal in the C-terminal region flanking the transmembrane domain [35].

### 2.3. The MARCH Family Members

There are only 11 proteins containing the RING-CH finger domain in humans and they are all contained in the MARCH family [36]. As the name membrane-associated RING-CH suggests, the MARCH proteins are integrated into the cellular membrane, with the exception of the two members MARCH7/RNF177 and MARCH10/RNF190, which have no transmembrane domain [37,38]. Two-transmembrane type MARCH proteins contain putative PDZ domain-binding motifs at their C-termini (type I: S/T-X-Φ; type II; Φ-X-Φ; type III; D/E-X-Φ; type IV; X-Ψ-D/E, where Φ and Ψ represent hydrophobic and aromatic residues, respectively) [39]. PDZ domains are protein–protein interaction modules often found in scaffold proteins. Binding to the PDZ domain facilitates the trafficking, localization and assembly of membrane proteins such as receptors, ion channels and transporters. The PDZ-binding motif is also found in several other transmembrane RNF proteins [19]. The N-terminal regions of MARCH4/RNF174, MARCH9/RNF179 and MARCH11 contain proline-rich sequences, which mediate protein–protein interactions (Figure 3) [40].

### 2.4. The RBR Family

The RBR family is characterized by the RBR signature, which consists of two RNFs linked by an in-between-ring (IBR) domain (Figure 3) [41]. While the N-terminal RNF (RING1 or N-RING) forms a canonical cross-brace structure, the C-terminal RNF (RING2 or C-RING) has a different tertiary structure, binding one metal ion [42]. This structural difference suggests that the two RNF domains are functionally different. The IBR domain comprises two zinc-binding structures in a C_6_HC configuration. It is established that the RING1 domain is a central hub of the E3 Ub ligase activity. In some cases, the IBR and RING2 are required for the correct E3 functions by their role in mediating protein–protein interactions with E2 enzymes and substrate proteins [41]. A large number of RBR proteins are found in eukaryotes from yeast to humans that have a diverse range of biological functions, including protein quality control, signaling, cell cycle and apoptosis [41]. Among the human RBR proteins, 5 proteins (Dorfin/RNF19A, RNF19B/IBRDC3, RNF144A, RNA144B/IBRDC2/p53RFP and RNF217/IBRDC1) have hydrophobic regions (Figure 3), but their membrane insertion sites have not yet been determined.

## 3. Cellular Functions of the Transmembrane RNF Proteins

### 3.1. Protein Degradation at the Endoplasmic Reticulum

The endoplasmic reticulum (ER) is an interconnected network of membranous tubes, vesicles and sacs, which carries out the synthesis, assembly and processing of secreted proteins and integral membrane proteins. Approximately 30% of the genes in eukaryotes encode secreted and transmembrane proteins [43,44,45]. Nascent polypeptide chains are co-translationally translocated across the ER membrane through the Sec61 translocon into the lumen and, in the case of transmembrane proteins, inserted into the ER membrane [46]. Subsequent folding and assembly are assisted by molecular chaperones and folding enzymes. Nevertheless, protein folding is an intricate and error-prone process that often results in misfolding. Impaired protein folding is also caused by genetic mutations as well as cellular stresses, such as heat shock and oxidative stress. Improperly folded proteins are retained in the ER and their accumulation induces stress in the ER. Increased ER stress induces cellular dysfunction and cell death. Cells are equipped with an evolutionarily conserved protective mechanism that reduces further accumulation of misfolded proteins, which is referred to as the unfolded protein response (UPR). The UPR includes (1) attenuation of protein translation, (2) degradation of ER-associated mRNA, (3) transcriptional induction of chaperones to increase the capacity of protein folding and (4) degradation of misfolded proteins by ER-associated degradation (ERAD) [47,48].

ERAD serves to eliminate misfolded proteins by targeting them for proteasomal degradation [49]. ERAD substrates also include native ER proteins, such as hydroxymethylglutaryl-CoA reductase (HMGR) and inositolo 1,4,5-triphosphate (IP_3_) receptors (IP_3_R), indicating that ERAD participates in both metabolism and intracellular signaling [50,51]. Proteins undergoing ERAD are transported back through the Sec61-based protein channel into the cytosol, where they are subjected to ubiquitination [52,53,54,55,56]. Poly-Ub tags on ERAD substrates are recognized by a cytosolic multimeric complex composed of the AAA-ATPase valosin-containing protein (VCP)/p97, Ufd1 and Npl4 (Cdc48p, Ufd1p and Npl4p in yeast). The VCP complex drives extraction of ERAD substrates from the ER and escorts them to the proteasome [57,58,59,60,61,62]. In yeast, at least three ERAD pathways (ERAD-C, ERAD-M and ERAD-L) have been proposed. ERAD-L degrades luminal proteins, and ERAD-C and ERAD-M recognize cytoplasmic and membrane domains, respectively [63,64,65,66]. Yeast contains two ER-resident E3 enzymes involved in three ERAD pathways: Hrd1p for ERAD-L and -M and Doa10p for ERAD-C [67,68,69,70]. In mammals, the ERAD system is conserved but more complicated, as reflected by the increased number of ER-localized E3 enzymes, which has been extensively reviewed elsewhere [71,72,73].

HRD1/Synoviolin, a mammalian ortholog of Hrd1p, is an ER-resident E3 enzyme with multiple transmembrane segments [74,75]. HRD1 forms a complex with the adaptor protein SEL1L, which recruits the ERAD machinery, including the E2 enzyme Ubc7, HERP, OS9, XTP3 and the Derlin proteins [75,76,77,78,79,80,81,82,83]. OS9 and XTP3 are ER luminal proteins containing mannose 6-phosphate receptor (MPR) homology domains, and they are required for, at least, the recognition of misfolded luminal glycoproteins [76,77,83]. The Derlins facilitate retrotranslocation of ERAD substrates [78,79,84]. Gp78/AMFR/RNF45 has a moderate level of sequence homology in the trasnsmembrane domains with HRD1. Gp78 associates with the E2 enzyme Ubc7 through the C-terminal CUE domain [85,86]. RMA1/RNF5 is a C-terminally anchored ER protein that associates with the E2 enzyme Ubc6 and Derlin-1 [87,88]. Numerous studies have demonstrated that HRD1, gp78 and RMA1 play an integral role in the ERAD pathway by mediating the ubiquitination of misfolded and native ER proteins, including cystic fibrosis transmembrane conductance receptor (CFTR), HMGR and CD3δ [74,75,85,86,87,88,89,90,91]. RMA1 cooperates with gp78 to target mutant CFTR for ERAD [92]. MARCH6/TEB4/RNF176, a mammalian ortholog of Doa10p, is a 14-transmembrane ER protein that catalyzes Lys48-linked ubiquitination with Ubc7 [70,93,94], but its precise biological function is less clear. The possible involvement of MARCH6 in the ERAD pathway in liver disease and metabolism has been suggested by recent studies demonstrating that MARCH6 ubiquitinates type 2 iodothyronine deiodinase (DIO2) and the mutant bile salt pump (Bsep)/ABCB11 associated with familial intraheoatic cholestasis type I for proteasomal degradation [95,96].

Kf-1/RNF103 was identified as a gene highly expressed in the cerebral cortex of Alzheimer disease patients [97]. Kf-1 has E3 activity and interacts with Derlin-1 and VCP [98]. *Kf-1* mRNA expression is predominantly detected in the hippocampus and cerebellum, and is elevated in the frontal cortex of rats after antidepressant treatment [97,99,100]. *Kf-1* knockout mice exhibit increased anxiety behavior [101]. Kf-1 may be involved in the regulation of neuronal activity and homeostasis in the central nervous system.

RFP2 is associated with VCP and Derlin-1, and regulates the turnover of CD3δ and the L-type calcium channel by mediating their ubiquitination [102,103]. ZNRF4 is also proposed to be an ER E3 enzyme that regulates the UPR and ERAD by controlling the stability of the ER chaperone calnexin [21].

Cellular cholesterol homeostasis is maintained through the actions of the sterol response element binding proteins (SREBPs), SREBP cleavage-activated protein (SCAP) and insulin-inducing gene (INSIG) [104]. SREBPs are synthesized as membrane proteins and bind to SCAP at the ER. Under low cholesterol conditions, SCAP escorts SREBPs to the Golgi where SREBPs are processed to release their N-terminal regions that act as transcription factors. Cleaved SREBPs enter the nucleus and transactivate genes related to choresterol and fatty acid metabolism. In contrast, high cholesterol conditions cause a conformational change in SCAP, which allows SREBP precursors to reside in the ER through their binding to the ER membrane protein INSIG [104]. Translocation in renal carcinoma, chromosome 8 gene (TRC8)/RNF139 was identified as a tumor suppressor gene product associated with renal carcinoma [105,106]. TRC8 is an ER protein with multiple transmembrane regions, including a sterol-sensing domain [106,107]. TRC8 is rapidly degraded by self-ubiquitination in the presence of sterols, while it becomes stable in the absence of sterols [107]. TRC8 interacts with SREBP-2 and SCAP. This interaction inhibits ER–Golgi transport and proteolytic processing of SREBP-2, thereby preventing SREBP-2 target gene expression [107]. TCR8 ubiquitinates INSIG-1, and probably SREBPs, thereby reducing their expression by proteasomal degradation [108]. Thus, TRC8 contributes to lipid metabolism by controlling the stability and trafficking of SREBPs.

RNF170 is implicated in the regulation of calcium signaling via the activity of IP_3_R. RNF170 is stably associated with the ER lipid raft proteins, erlin1 and erlin2, that bind to IP_3_R upon cell activation. Through this interaction, RNF170 recognizes and ubiquitinates activated IP_3_R to target it for ERAD [109]. RNF170 also has pathogenic importance, since a mutation in the *RNF170* gene is associated with autosomal dominant sensory ataxia [110]. Forced expression of zebrafish RNF170 with the mutation causes failure in embryonic development in zebrafish, suggesting that RNF170 is involved in neuronal transmission and differentiation [110].

The calcium sensing receptor (CaR) is a member of the G protein-coupled receptor family, and has an important role in calcium homeostasis through its effect on regulating parathyroid hormone secretion and renal calcium reabsorption [111]. The Dorfin E3 enzyme interacts with CaR through their C-termini, and this promotes ubiquitination of CaR for ERAD targeting [112]. The precursor forms of CaR, which reside in the ER, are more sensitive to Dorfin-mediated ubiquitination than mature CaR, suggesting that Dorfin controls the protein levels of active CaR during protein synthesis [112]. Overexpressed Dorfin is accumulated in an aggresome-like structure near the centrosome, which is characteristic of a variety of neurodegenerative diseases [113]. Dorfin is present in Lewy body-like inclusions in neurons from familial and sporadic amyotrophic lateral sclerosis (ALS) and Parkinson disease [113]. Dorfin ubiquitinates mutant Cu/Zn-superoxide dismutase-1 (SOD1) and accelerates its degradation, which in turn reduces protein aggregation and cell toxicity [113,114,115,116]. The biological features and association with neurodegenerative diseases suggest that Dorfin protects the nerve system by mediating protein quality control.

Bifunctional apoptosis regulator (BAR)/RNF47, a member of the B cell lymphoma (Bcl)-2 family, was originally identified as a regulator of apoptosis (see Section 3.3). BAR is an ER-resident E3 enzyme that interacts with Ubc7, and is itself an ERAD substrate that undergoes self-ubiquitination [117]. However, BAR is unlikely to be a central mediator of ERAD since it does not affect the turnover of typical ERAD substrates, including T cell receptor (TCR)α and CD3δ [117]. Rather, BAR contributes to the cellular adaptation to ER stress by regulating inositol-requiring protein-1 (IRP1) signaling, one of the UPR signaling pathways. IRP1 is an ER-anchored protein that mediates alternative splicing of the transcription factor XBP1 under ER stress conditions, which induces the transcriptional upregulation of chaperones and ERAD components [118,119,120]. Activated IRP1 interacts with the signaling adaptor tumor necrosis factor (TNF) receptor-associated factor (TRAF)2 and then activates downstream kinases such as the c-Jun N-terminal kinases (JNKs) [121]. BAR ubiquitinates Bax inhibitor-1 (BI-1), an ER-resident negative regulator of IRE1, targeting the protein for proteasomal degradation [117]. Depletion of BAR expression increases the BI-1 protein level, thereby suppressing IRE1 activity [117]. BAR thus controls directly cell viability by regulating UPR as well as apoptosis.

RNF122 is also very unstable due to its self-ubiquitination, but is stabilized by the interaction with calcium-modulating cyclophilin ligand (CAML), an ER transmembrane protein implicated in calcium signaling [122]. This interaction is likely to be mediated through the RNF domain [122], suggesting that CAML may interfere with RNF122 E3 activity. RNF122 may regulate calcium signaling at the ER.

Rines/RNF180 is a tail-anchored ER E3 enzyme highly expressed in the brain [123]. Through the basic coiled-coil domain, Rines interacts with the transcription factor Zic2, which is associated with holoprosencephaly, a congenital malformation of the forebrain. Overexpressed Rines promotes ubiquitination and proteasomal degradation of Zic2 in cultured cells. However, the Zic2 protein levels are not significantly altered in the brain of *Rines* knockout mice. The precise *in vivo* function of Rines is therefore still uncertain.

### 3.2. Cell Proliferation and Differentiation

RNF13, a member of the PA-TM-RING family, appears to be associated with cell proliferation, differentiation and tumorigenesis [30]. RNF13 was found to be highly expressed in chicken skeletal myoblasts [124]. The expression of RNF13 gradually decreases during skeletal myogenesis, and is upregulated by myostatin, a myogenesis negative regulator. Overexpression of RNF13 inhibits skeletal muscle proliferation in a manner that is dependent on its E3 activity. Thus, RNF13 may contribute to myogenesis as a negative regulator of cell proliferation. In addition, RNF13 expression is increased in differentiating rat B35 neuroblastoma cells, and overexpression of RNF13 promotes neurite extension in rat PC12 pheochromocytoma cells, suggesting an involvement of RNF13 in neuronal development [125]. High-level expression of RNF13 is also observed in pancreatic ductal adenocarcinoma as well as in precancerous pancreatic lesions, suggesting its involvement in cancer development [126]. RNF13 has been reported to be localized to various membrane structures, including the nucleus, ER, Golgi apparatus and endosomes. Recent studies have reported that RNF13 is present in the endosomal and lysosomal compartments, and the C-terminal region containing the RNF domain is released into the cytosol by proteolytic cleavage [125]. Activation of protein kinase C inhibits this cleavage and stabilizes the full-length RNF13 [127]. The stabilized RNF13 is then transported to the inner nuclear membrane via the recycling endosomes, thereby exposing its RNF domain to the nucleoplasm. RNF13 may modulate gene expression and signal transduction by mediating ubiquitination in response to various stimuli [35,127]. It is imperative to identify the RNF13 substrate proteins to obtain a better understanding of the physiological role and mechanism of action of RNF13.

Spermatogenesis is a developmental process of male germ cells that is divided into three phases in mammals: (1) self-renewal and mitotic proliferation of spermatogonial stem cells, (2) meiosis of spermatocytes, and (3) metamorphosis of haploid round spermatids into flagellated spermatozoa, including acrosome and flagellum formation, mitochondrial rearrangement and nuclear elongation and condensation (spermiogenesis). The complicated process of spermatogenesis requires a unique mechanism regulating, for instance, gene expression, chromatin condensation, protein transport, signal transduction and protein degradation. Almost 4% of the genes in the mouse genome are estimated to be male-germ-cell specific [128], and a number of testis-specific RNF proteins have been identified which are thought to play a role in spermatogenesis and fertilization. RNF133, a member of the PA-TM-RING family, is an E3 enzyme that is highly and specifically expressed in male germ cells undergoing spermiogenesis (spermatids) [129]. RNF133 is localized to the ER in the immortalized mouse spermatocyte cell line GC-2 and catalyzes self-ubiquitination in 293T cells, suggesting that RNF133 is involved in ER protein quality control during spermatogenesis. The expression of Dorfin is increased in male germ cells during spermatogenesis [130]. In rat spermatids, Dorfin is localized to the Golgi apparatus, acrosomal membrane and tail, and interacts with the 26S proteasome subunit Psmc3. Dorfin may be associated with the UPS in shaping the spermatid head and tail. MARCH11 is another male germ cell-specific E3 enzyme, which is described in section 3.6.

### 3.3. Apoptosis

Apoptosis is a complex, highly regulated cell-death process, which is mediated through extrinsic and intrinsic signal pathways. The extrinsic pathway is initiated by activation of the TNF family death receptors, such as Fas/CD95, TNFα receptor and TNF-related apoptosis-inducing ligand (TRAIL) receptor. Stimulation of the death receptors by ligand binding allows them to interact with the cytoplasmic adapter molecules, such as Fas-associated death domain (FADD) and TNF receptor-associated death domain (TRADD), through the cytoplasmic death domains. The death effecter domains (DEDs) of FADD and TRADD then recruit the DED-containing caspases, caspase-8 and caspase-10, which activate downstream caspases and proteases to induce apoptosis [131]. The intrinsic pathway is stimulated by various cellular stresses, including ER stress, oxidative stress, DNA damage and growth factor deprivation. These stimuli increase mitochondrial membrane permeability, leading to leakage of cytochrome *c* into the cytosol. This cytochrome *c* release activates caspases through binding to apoptosis protease activating factor-1 (Apaf-1) [132]. Members of the Bcl-2 family play important roles in the regulation of mitochondria-dependent apoptosis in both the extrinsic and intrinsic pathways. The pro-apoptotic Bcl-2 family members, Bax and Bak, are essential mediators of mitochondrial membrane permeability, whereas the anti-apoptotic members, Bcl-2 and Bcl-x_L_, bind to the pro-apoptotic proteins and hinder apoptosis [132,133].

BAR was identified as an inhibitor of Bax-induced apoptosis and is an ER-localized E3 enzyme predominantly expressed in neurons of the central nervous system [134,135]. Overexpression of BAR results in a protection of neural cells from apoptosis, whereas knockdown of BAR increases apoptosis [135]. The anti-apoptotic effect of BAR requires the transmembrane, sterile alpha motif (SAM) and DED-like domains, but not the RNF domain, suggesting that BAR E3 activity is not involved in the suppression of cell death [135]. The SAM domain binds to Bcl-2 and Bcl-x_L_, thereby interfering with Bax-induced apoptosis. On the other hand, the DED-like domain interacts with caspase-8 and caspase-10, thereby inhibiting Fas-induced apoptosis [135]. Thus, BAR protects neuronal cells from apoptosis that is mediated by either the extrinsic or intrinsic pathway.

The p53 tumor suppressor is known to induce apoptosis in response to oncogenic stress through the transactivation of apoptotic genes, including Bax [136]. Upon apoptotic stimuli, p53 promotes the expression of the RNF144B E3 enzyme, which interacts with the cell cycle regulator p21/WAF [137]. RNF144B may promote the ubiquitination and degradation of p21/WAF, thus facilitating a shift of cellular response from growth arrest to p53-mediated apoptosis [137,138]. However, a recent study by Sayan *et al.* [139] reported that RNF144B expression is upregulated by the p53-related transcription factor p73, but not by p53. There are two *p73* isoforms encoding the TAp73 and ΔNp73 proteins with or without the transactivation domain, respectively. TAp73 is able to induce apoptosis, while ΔNp73 blocks p53- and p73-mediated transactivation [140,141]. In response to DNA damage, cells preferentially degrade ΔNp73, preventing its inhibitory effect and promoting TAp73-mediated apoptotic processes [142]. RNF144B targets both the p73 isoforms for ubiquitnation, but ΔNp73 is degraded faster than TAp73. As a result, the relative levels of TAp73 are increased, allowing TAp73 to exert its pro-apoptotic activity [142]. In the steady state, RNF144B is localized mainly in the cytosol [137,139,143], but it is translocated to mitochondria during apoptosis [143]. This translocation requires the putative transmembrane domain of RNF144B and activated mitochondrial Bax [143]. RNF144B interacts with activated Bax and influences Bax ubiquitination and stability. Knockdown of RNF144B results in increased Bax levels and enhances cell sensitivity to apoptosis [143]. Although the precise function and regulatory mechanism of RNF144B has not been elucidated, the role of RNF144B in apoptosis is evident.

RNF182 appears to be a pro-apoptotic factor. RNF182 was identified as a brain-specific E3 enzyme that targets ATP6V0C, a component of vacuolar ATPase, for proteasomal degradation [144]. Its mRNA levels are elevated in the brain with Alzheimer disease and in NT2 neuron cells undergoing stress-induced apoptosis. Increased RNF182 expression makes the neuron cells highly sensitive to stress-induced apoptosis [144]. Although the downregulation of ATP6V0C is unlikely to contribute to neuronal apoptosis, it may facilitate the regulation of pH homeostasis in neuronal cells.

RFP2 also appears to be involved in the induction of apoptosis [145]. UV irradiation stabilizes the RFP2 protein in HEK293 human embryonic kidney cells, leading to increased ubiquitination of the anti-apoptotic proteins Akt kinase and Mdm2, a cytosolic E3 enzyme of p53. Proteasomal degradation of Mdm2 and Akt enhances ionizing radiation-induced apoptosis by increasing p53 stability and by decreasing anti-apoptotic Akt signaling [145].

An increasing body of evidence indicates that breakdown of the lysosomal membrane triggers apoptosis. Increased lysosomal permeability results in a release of lysosomal proteases into the cytosol, and the released proteases stimulate the mitochondria-dependent apoptotic pathway with increased mitochondrial membrane permeability [146]. RNF152 is a C-terminally anchored E3 enzyme that undergoes proteasomal degradation by Lys48-linked self-ubiquitination [147]. Overexpressed RNF152 is localized to the lysosomes and has a pro-apoptotic activity in a manner dependent on its E3 activity, suggesting a possible role in lysosome-associated apoptosis [147].

### 3.4. Structural Integrity of the Golgi Apparatus

The Golgi apparatus is the processing and sorting site for newly synthesized proteins received from the ER. It consists of a stack of membrane cisternae, which is divided into the three functionally distinct *cis*-, *medial*- and *trans*-Golgi regions. The region between the ER and *cis*-Golgi apparatus contains numerous vesicular and tubular membrane structures called the intermediate compartment (IC) or ER-Golgi intermediate compartment (ERGIC). The ICs are transport intermediates between the ER and *cis*-Golgi apparatus, which are formed by homotypic fusion of ER-derived cargo vesicles. The ICs continually fuse with and pass along cargo proteins to the *cis*-Golgi apparatus. In addition, the ICs mediate retrograde transport of proteins returning from the Golgi to the ER [148]. The influx and efflux of lipids and proteins are indispensable to the organization of the Golgi apparatus. For example, when the ER-to-Golgi transport is blocked by brefeldin A, the Golgi proteins and membranes are redistributed into the ER and the Golgi apparatus eventually disappears [149]. Efficient IC-to-Golgi trafficking is in part achieved by coordinated action of the Golgi matrix proteins, including GM130, Golgi reassembly stacking protein (GRASP)65, p115 and giantin [150]. GM130, a long coiled-coil protein, is associated with the cytoplasmic surface of the *cis*-Golgi and IC membranes through binding of its C-terminal region to myristoylated GRASP65 [151,152]. The N-terminal region of GM130 interacts with the docking protein p115 that binds to the transmembrane Golgi protein giantin [153,154]. Moreover, GM130 and p115 associate with the soluble *N*-ethylmaleimide-sensitive factor attachment protein receptor (SNARE) proteins, which mediate membrane fusion [150,155]. The GRASP–GM130– p115–giantin complex forms a molecular bridge linking apposed membranes of the *cis*-Golgi apparatus and/or ICs, promoting their tethering and fusion.

Zinc finger protein-like 1 (ZFPL1) is a single transmembrane protein C-terminally anchored to the *cis*-Golgi membrane [156]. Its cytoplasmic N-terminal region contains a zinc finger motif and a variant RNF domain in which the fourth conserved histidine residue is substituted to aspartic acid. The zinc finger motif, but not the RNF domain, mediates the interaction of ZFPL1 with the C-terminal coiled-coil segment of GM130 [156]. Knockdown of ZFPL1 does not affect the protein levels of GM130, but delays the ER-to-Golgi trafficking, leading to a redistribution of GM130, p115 and GRASP65 into the ICs. A trafficking defect causes tubular elongation of the ICs and impaired reassembly of the *cis*-Golgi apparatus [156]. Thus, ZFPL1 is likely to provide a scaffold function for the *cis*-Golgi matrix proteins to assist membrane tethering and fusion. Interestingly, the RNF domain appears to be required for the ZFPL1 function. It is an important issue to address whether ZFPL1 behaves as an E3 enzyme in *cis*-Golgi organization and trafficking.

### 3.5. Downregulation of Cell-Surface Molecules—Immune Regulation

**Figure 4:**
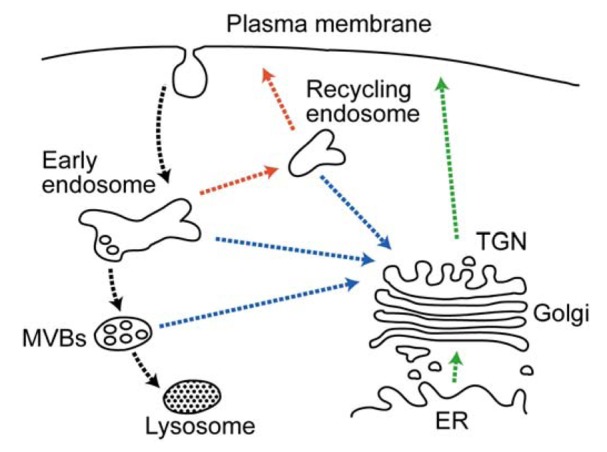
Schematic diagram of the organization of organelles and the intracellular transport pathways. The major membrane-bound organelles and the main routes of protein transport are indicated, with the secretory pathway in green, the endocytic degradation pathway in black, the recycling pathway in red and the retrograde pathway in blue.

The expression patterns and functions of plasma membrane proteins, such as receptors, transporters and channels, are regulated by endocytosis, which is triggered, in general, by ubiquitination [157]. Endocytosed proteins are delivered to the peripheral population of endosomes, which are referred to as either early or sorting endosomes, where the proteins are sorted into two classical trafficking routes: the degradation pathway destined for the lysosomes via the late endosomes and the recycling pathway back to the plasma membrane via the recycling endosomes (Figure 4). Proteins en route to the lysosomes are sorted to the multivesicular bodies (MVBs) in the early endosomes. In this sorting step, the cargo proteins on the endosomal limiting membrane are incorporated into the invaginating membrane destined to form the intraluminal vesicles of the MVBs. Eventually, the MVBs fuse with the late endosomes and lysosomes, thereby degrading the intraluminal vesicles [158]. Lys63-linked Ub chains have been shown to play a role in MVB sorting [159], which is mediated by a series of cytosolic protein complexes termed the endosomal sorting complex required for transport (ESCRT) [160,161]. The Ub tag is first recognized by ESCRT-0, which triggers sequential recruitment of the ESCRT-1, ESCRT-2 and ESCRT-3 complexes. The ESCRT-3 complex forms polymeric filamentous structures to generate an invaginating membrane bud, in which cargo proteins are incorporated [161].

Recent studies have demonstrated that MARCH proteins have important roles in immune regulation by controlling the endocytic downregulation of cell-surface immune molecules [36,162]. The role of MARCH proteins in immune regulation was first reported by Goto *et al.* [163] in 2003, demonstrating that the overexpression of MARCH8/RNF178 (originally termed cellular modulator immune regulation (c-MIR)) induces ubiquitination and endocytic degradation of the costimulatory molecule CD86/B7-2. Bartee *et al.* [164] has reported that other two-transmembrane type MARCH proteins also have similar effects that downregulate the cell-surface expression of immune regulators, such as CD86, major histocompatibility complex (MHC) class I (MHC I), Fas and intracellular adhesion molecule (ICAM)-1.

MARCH8 is specifically expressed in the lymph nodes, spleen and antigen-presenting cells (APCs), such as dendritic cells (DCs) and monocytes [163]. Transgenic mice, in which MARCH8 is overexpressed in APCs, are impaired in antigen presentation and T cell development, conferring marked resistance to the onset of experimental autoimmune encephalomyelitis [165]. As would be expected from these observations, MARCH8 targets MHC class II (MHC II) for ubiquitination, leading to rapid endocytosis and lysosomal degradation [165]. Although the exact subcellular localization of endogenous MARCH8 is obscure, a recent study using a chimeric CD86 protein has demonstrated that ubiquitination occurs upon the interaction between MARCH8 and substrate proteins in the plasma membrane [166].

MARCH1/RNF171 has a high degree of sequence homology with MARCH8 and is expressed in the lymph nodes and spleen, as well as being highly expressed in B cells and moderately in DCs and macrophages [167], suggesting a similar physiological function to MARCH8. Indeed, MARCH1 shares ubiquitination substrates with MARCH8 (*i.e.*, MHC II and CD86) [167-169]. In B cells from *March1* knockout mice, MHC II is less ubiquitinated and more stable [167]. As a result, the levels of cell-surface MHC II are remarkably elevated and the antigen-presenting ability of B cells is enhanced. A high level of expression of MHC II also occurs in the DCs of *March1* knockout mice [170,171]. There is a close correlation between the cell surface expression of MHC II and DC maturation. MHC II is ubiquitinated and rapidly degraded in immature DCs, while it is stably expressed on the cell surface after maturation [172,173]. Since MARCH1 is a short-lived protein targeted to lysosomal degradation [174], its protein expression is susceptible to a change in its message levels. Both the mRNA and protein expression of MARCH1 are specifically suppressed during the maturation of DCs, which is likely to stabilize MHC II due to the low level of ubiquitination [175]. The stability of CD86, which is critical for DC-mediated activation of T cells, is also affected by MARCH1 expression. DCs from *March1* knockout mice exhibit increased surface expression of CD86 and fail to activate T cells [170]. MARCH1-mediated ubiquitination of CD86 is essential for both the protein turnover of CD86 in DCs and DC-mediated T cell activation [168].

Recent studies have indicated that interleukin (IL)-10 tightly regulates the MARCH1-mediated ubiquitination of MHC II and CD86 [168,176]. IL-10 is a potent immunosuppressive cytokine that downregulates the surface expression of MHC II and CD86 in DCs [177]. When DCs are stimulated by lipopolysaccharide (LPS), the mature DCs induce MHC II and CD86 expression. LPS-stimulated DCs, however, produce IL-10 that acts in an autocrine manner to limit the surface levels of MHC II and CD86, thereby ameliorating excessive immune and inflammatory responses [178]. IL-10 has been shown to promote *MARCH1* transcription [179]. In LPS-stimulated DCs, autocrine IL-10 increases the protein levels of MARCH1 and lowers the surface levels of MHC II and CD86 due to the high level of MARCH1-mediated ubiquitination [168,176]. Moreover, MARCH1 activity is also controlled at the post-translational level. In mature DCs, CD83, a transmembrane glycoprotein of the immunoglobulin superfamily, interacts with MARCH1 and blocks the binding of MARCH1 to MHC II and CD86, thereby interfering with their ubiquitination [176]. Taken together, MARCH1 and MARCH8 are essential regulators of the immune response, controlling the stability of MHC II and CD86 in APCs.

MARCH4 and MARCH9 have also been thought to be involved in immune regulation by downregulating MHC I, CD4, ICAM-1, Mult1 (a ligand of the natural killer cell receptor NKG2D) and several B-cell proteins, but less is known about their precise physiological functions [164,180,181,182]. MARCH4 catalyzes Lys63-linked polyubiquitination of CD4, which targets the protein for lysosomal degradation [183]. In addition, MARCH8 forces a change on the trafficking route of clathrin-independent cargo, such as CD44 and CD98, from the recycling pathway to the MVB–lysosomal pathway [184]. These facts suggest that MARCH-mediated ubiquitination affects the stability of cell-surface transmembrane proteins by controlling endosomal sorting as well as endocytosis.

T cell anergy (unresponsiveness) is a mechanism of peripheral tolerance to prevent an inappropriate immune response to self-antigens and/or harmless environmental antigens [185,186]. T cell activation requires coordinate signaling from both TCR and co-stimulatory molecules such as CD28. TCR engagement without co-stimulation initiates T cell anergy. Anergic T cells are unable to proliferate and produce IL-2. GRAIL is a ubiquitously expressed endosomal E3 enzyme implicated in inducing T cell anergy [187,188,189]. The mRNA and protein expression of GRAIL are tightly regulated in T cells [187,188]. GRAIL expression is up-regulated in T cells during anergy induction and this inhibits T cell proliferation and cytokine production [187]. This inhibitory effect results from a blocking of the co-stimulatory signaling, which is accompanied by GRAIL-mediated ubiquitination and subsequent downregulation of cell-surface CD154, CD83, CD81, CD151, Rho GDP-dissociation inhibitor (RhoGDI) and CD3ζ [33,34,190,191,192]. Disruption of the *Grail* gene causes increased susceptibility to experimental autoimmune encephalitis in mice, in which T cells exhibit hyperproliferation and overproduction of cytokines upon either TCR stimulation or co-stimulation [192]. The loss of GRAIL causes increased surface expression of the TCR–CD3 complex on naϊve T and regulatory T (Treg) cells [192]. Thus, GRAIL has an important role in T cell activation and differentiation, as well as anergy, by controlling the TCR and co-stimulation signaling pathways.

### 3.6. Membrane Trafficking Between the Trans-Golgi Network (TGN) and Endosomes

Several classes of endocytosed proteins follow the retrograde transport pathway to the TGN from the endosomes (Figure 4) [193]. TGN and endosomal receptors and processing enzymes, such as MPRs and the furin peptidase, are known to cycle between the endosome/TGN and plasma membrane. The recycling of these proteins facilitates the delivery of lysosomal enzymes and maintains the processing capacity of the TGN. In addition, bacterial and plant protein toxins (e.g., Shiga, Cholera and ricin toxins) and viral proteins utilize the retrograde pathway to inflict cell damage and induce cytotoxicity and pathogenicity. The docking and fusion of transporting vesicles to their target membranes are controlled by a complex array of cellular proteins, including Rab and ADP-ribosylation factor (Arf) small GTPases and SNAREs. Membrane fusion is mediated by the formation of a SNARE complex between a vesicle known as a (v)-SNARE (also termed Q-SNARE) on the transporting vesicle and target (t)-SNAREs (also termed R-SNAREs) on the target membrane, with the aid of Rab GTPases [194]. The early/recycling endosome-to-TGN (EE/RE–TGN) transport requires the SNARE complex, which is comprised of the t-SNAREs syntaxin-6, syntaxin-16 and Vti1a and the v-SNARE VAMP3 or VAMP4, as well as the Rab6 and Rab11 GTPases. On the other hand, the late endosome-to-TGN transport utilizes the SNARE complex comprised of syntaxin-10, syntaxin-16 and Vti1a along with Rab9 [195,196].

MARCH2/RNF172 is an endosomal E3 enzyme that interacts with syntaxin-6 through its C-terminal cytoplasmic tail [197]. Knockdown of MARCH2 results in a redistribution of syntaxin-6 from the TGN to the plasma membrane, and perturbs the trafficking of TGN38 and TGN46, with these TGN proteins cycling between the TGN and plasma membrane via the early/recycling endosomes [197]. The effect of MARCH2 knockdown appears to be specific to the EE/RE–TGN pathway, since the localization and retrograde transport of furin, a TGN protein which cycles between the TGN and plasma membrane via the late endosomes, is not affected. The overexpression of MARCH2 induces the accumulation of syntaxin-6 and its SNARE partners (Vti1a and VAMP3) in the endosomal compartments, where several recycling proteins, including TGN38, furin and the transferrin receptor, are trapped [197]. Thus, MARCH2 is likely to be involved in at least the regulation of EE/RE–TGN transport, but the functional significance of its binding to syntaxin-6 and its E3 activity remains unknown. MARCH3/RNF173, the closest homolog of MARCH2, is also localized to a subset of the early endosomes has functional properties similar to MARCH2 [198]. Since MARCH3 binds to MARCH2 as well as syntaxin-6, MARCH3 may cooperate with MARCH2 to function in the EE/RE–TGN pathway. The subcellular localization of MARCH2 and MARCH3 depends on the PDZ-binding motifs in their C-termini. Deletion of the PDZ motif results in mislocalization of MARCH2 and MARCH3 to the ER [197,198]. Another study demonstrated that the MARCH2 PDZ motif interacts with human Discs Large 1 (hDLG1)/SAP97, a multiple-PDZ scaffold protein involved in the formation of cell–cell junctions in epithelial cells [19]. This interaction facilitates the stable recruitment of MARCH2 to the cell–cell contact sites [19]. Moreover, MARCH2 targets hDLG1 for ubiquitination, which may affect the stability of the hDLG1 localization at the cell–cell contact sites [19]. It is possible that MARCH2 plays a role in maintaining cell polarity and epithelial integrity as well as endosomal trafficking.

The role of the MARCH family in membrane trafficking has also been suggested by the finding that the MARCH11 E3 enzyme is specifically expressed in the early developmental stages of spermatids [199]. Immunoelectron microscopy revealed that MARCH11 is localized to the TGN-derived vesicles and MVBs. In these membrane compartments, MARCH11 associates with ubiquitinated fucose glycoproteins and the adaptor protein complex (AP)-1 [199]. In somatic cells, AP-1 mediates protein sorting between the TGN and endosomes [200]. In early spermatids, the secretory pathway is specialized for biogenesis of the acrosome, an enzyme-filled membrane sac that is essential for the ovum [201]. Numerous TGN-derived vesicles fuse with one another to form the immature acrosomal vesicle attached to the nucleus. Continuous vesicle fusion allows an increase in the size of the acrosome and for it to become filled with hydrolases, including lysosomal enzymes. However, not all newly synthesized proteins are incorporated into the acrosome, and some proteins are delivered to the MVBs. These proteins include the fucose glycoproteins and several endosomal and lysosomal proteins, such as MPRs and lysosome-associated membrane proteins (LAMPs) [202,203]. Although it has been shown that post-Golgi trafficking is essential for acrosome formation [204,205], the mechanism underlying protein sorting and targeting is much less well understood. In somatic cells, a Ub conjugation is recognized as a sorting signal that directs the incorporation of ubiquitinated proteins into transporting vesicles at the TGN [206,207]. In early spermatids, ubiquitinated proteins are enriched in the TGN-derived vesicles and MVBs, and the ESCRT machinery STAM2 and Hrs are present in the early endosomes [208,209]. Thus, male germ cells possess a Ub-dependent sorting mechanism which allows them to be conducted in the TGN-to-MVB transport pathway. Both the localization and AP-1 binding suggest that MARCH11 participates in protein sorting at the TGN and endosomes through a mediation of the ubiquitination of cargo proteins.

### 3.7. Mitochondrial Morphology and Function

Mitochondria are organelles crucial to a variety of cellular functions, including energy production, catabolism and anabolism, thermogenesis, calcium homeostasis and apoptosis. Mitochondria form a dynamic network, which is tightly maintained by a balance between opposing fusion and fission events. This dynamic behavior is important for both the structural and functional integrity of mitochondria [210]. For example, mitochondrial fusion protects mitochondrial function by allowing a mixing of mitochondrial contents so as to minimize the accumulation of damaged mitochondrial DNA and proteins. There is evidence that mtDNA mutations are accumulated in age-related neurodegenerative diseases, which emphasizes the close link of mitochondrial dynamics to both aging and pathogenesis. Since the first identification of the gene product responsible for mitochondrial fusion in *Drosophila* [211], the key regulators of mitochondrial fusion and fission have been identified and extensively characterized in yeast and mammals [212,213,214,215,216,217,218,219,220]. In mammals, mitochondrial fusion is mediated by the dynamin-related GTPases mitofusin (MFN)1 and MFN2 of the mitochondrial outer membrane (MOM) [220], and optic atrophy 1 (OPA1) of the mitochondrial inner membrane and intermembrane spaces [219]. MFN1 and MFN2 mediate MOM fusion, while OPA1 mediates mitochondrial inner membrane fusion. Mice deficient in both *Mfn1* and *Mfn2* are embryonically lethal [221]. Mutations in MFN2 and OPA1 are associated with human neuropathies, such as Charcot-Marie-Tooth disease type 2A (CMT2A) and dominant optic atrophy (DOA), respectively [222,223,224]. Mitochondrial fission is controlled by the cytosolic GTPase dynamin-related protein (DRP)1 [212]. DRP1 assembles around the site of fission to form a contractile ring and divides mitochondria in a GTP-dependent manner. Targeted disruption of *DRP1*, results in severe abnormalities in brain development and embryonic lethality in mice [225,226]. Given the developmental and pathological importance of the mitochondrial fusion and fission factors, mitochondrial dynamics is a field of research of considerable interest, but the mechanistic details of the mitochondrial dynamics and the relevant cellular functions are still an open question at present.

The involvement of the UPS in mitochondrial dynamics was first suggested by genetic studies in budding yeast, demonstrating that impaired mitochondrial morphology and inheritance were caused by disruption of components of the UPS [227,228]. Mdm30p, a component of the Skp1-Cullin-F-box (SCF) Ub ligase complexes, is proposed to regulate mitochondrial fusion mediated by Fzo1p, the yeast ortholog of MFNs [229]. The loss of Mdm30p increases the protein levels of Fzo1p and causes mitochondrial aggregation [230]. In addition, the Mdm30p-associated SCF complex catalyzes Lys48-linked polyubiquitination of Fzo1p to target the protein for proteasomal degradation, which facilitates MOM fusion [231,232]. These findings provide evidence that mitochondrial dynamics are regulated by ubiquitination in yeast.

The role of ubiquitination in mammalian mitochondrial dynamics was uncovered by the identification of the mitochondrial E3 enzyme MARCH5/MITOL/RNF153 that is embedded in the MOM. Two earlier studies proposed that MARCH5 acts as a regulator of mitochondrial fission, as it ubiquitinates DRP1 and Fis1, a MOM-anchored protein involved in mitochondrial fission, and loss of its activity results in mitochondrial fragmentation [233,234]. One of the two studies showed that ubiquitination of DRP1 and Fis1 accelerates their protein turnover by proteasomal degradation [234]. However, the mechanism by which MARCH5 regulates DRP1 remains controversial. Karbowski *et al.* [235] have reported that loss of MARCH5 activity results in the formation of a highly interconnected mitochondrial network without affecting the protein levels of DRP1. This mitochondrial elongation is likely to be due to a decreased fission rate caused by increased recruitment and stabilization of DRP1 on mitochondria, suggesting that MARCH5 may regulate DRP1 activity by controlling its transition between the cytosol and mitochondria rather than by proteasomal degradation. MARCH5 appears to regulate mitochondrial fusion through an interaction with MFN1 and MFN2 [233,236]. A recent study has shown that MARCH5 promotes proteasomal degradation of MFN1 by ubiquitinating it, and knockdown of MARCH5 increases the MFN1 levels [236], which lends support to the notion proposed by Karbowski *et al.* In the MARCH5 knockdown cells, levels of reactive oxygen species are increased and the mitochondrial membrane potential is reduced, suggesting that the MARCH5 activity counteracts senescence [236].

It is considered that mitochondria contain a protein quality control system analogous to ERAD [237,238,239]. VCP, a critical mediator of ERAD, is recruited on mitochondria and contributes to proteasomal degradation of the MOM proteins MFN1, MFN2 and myeloid cell leukemia sequence-1 (MCL1) [237,239]. In addition, proteins of other mitochondrial compartments also undergo proteasomal degradation by an unknown mechanism [240]. It is important to determine whether and how MARCH5 is involved in mitochondrial protein quality control as in the case of the ERAD E3 enzymes. MARCH5 may recognize mitochondrial misfolded proteins and thus function in cell survival by acting against the toxicity of protein aggregation in mitochondria. Protein aggregation is acknowledged as a hallmark of neurodegenerative disorders such as Alzheimer's, Parkinson's, Huntington's, ALS and Machado-Joseph diseases. Although it is uncertain whether protein aggregation is a cause or consequence of each disorder, it induces cellular dysfunction and eventually cell death. Mutant SOD1 and mutant polyglutamine ataxin-3 are known to form toxic aggregates in mitochondria in ALS and Machado-Joseph disease, respectively [241,242]. MARCH5 selectively removes these mitochondria-associated aggregates by promoting their ubiquitination and proteasomal degradation, thereby reducing cell toxicity [243,244].

Mitochondria are involved in the antiviral immune response [245,246]. A recent study has provided interesting evidence that MARCH5 controls the signaling pathway in innate immunity. MARCH5 catalyzes Lys63-linked polyubiquitination of TRAF-associated NFκB activator (TANK)/I-TRAF, a TRAF-binding protein that inhibits Toll-like receptor (TLR) signaling [247]. TANK ubiquitination blocks the interaction between TANK with TRAF6, which stimulates TLR7-mediated NF-κB activation. Taken together, MARCH5 plays a pivotal role not only in mitochondrial dynamics, but also mitochondrial homeostasis and signaling.

**Figure 5:**
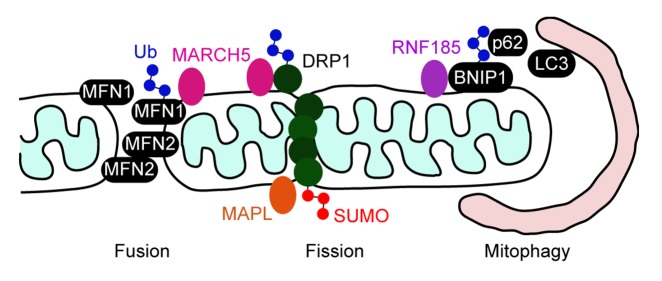
Proposed roles of the transmembrane RNF proteins in mitochondrial dynamics and mitophagy. MARCH5 may regulate mitochondrial fusion and fission by ubiquitinating MFN1 and DRP1. MAPL SUMO ligase stabilizes DRP1 and therefore promotes mitochondrial fission. RNF185-mediated ubiquitination triggers mitophagy.

Mitochondria-anchored protein ligase (MAPL)/MULAN was first identified as an E3 Ub ligase of the MOM that influences mitochondrial morphology, as its depletion causes mitochondrial clustering at the perinuclear region [20]. However, a recent report has demonstrated that MAPL catalyzes sumoylation, but not ubiquitination, under physiological conditions [248]. Furthermore, MAPL targets DRP1 for sumoylation and the SUMO modification stabilizes DRP1 at the sites of mitochondrial fission, leading to mitochondrial fragmentation, suggesting that MAPL is a SUMO ligase that regulates mitochondrial fission (Figure 5) [248]. MAPL is also localized to the small vesicles derived from mitochondria, which are targeted to the peroxisomes [249,250]. It is possible that MAPL shuttles between the mitochondria and peroxisomes to control the biogenesis and functions of the peroxisome.

Mitophagy is a homeostastic mechanism which selectively degrades damaged mitochondria [251]. Parkin, a cytosolic E3 enzyme, is recruited to damaged mitochondria through interaction with the PINK kinase of the MOM, and then ubiquitinates MOM proteins such as MFN2 and the voltage-dependent anion channel (VDAC1) [252,253,254,255]. The autophagy receptor p62 recognizes the ubiquitinated mitochondrial proteins and promotes autophagocytosis of damaged mitochondria through binding to LC3, an adaptor protein of the autophagosome [255]. Recently, RNF185 was identified as another MOM Ub ligase which mediates mitophagy independently of Parkin [256]. RNF185 activates LC3 and promotes autophagolysosome formation. RNF185 interacts with the Bcl-2 family member BNIP1 on mitochondria, and catalyzes Lys63-linked polyubiquitination of BNIP1. Subsequently, mitophagy is accomplished by the recruitment of p62 on the mitochondrial surface by its binding to ubiquitinated BNIP1 (Figure 5) [256].

Goliath has been shown to be present in the mitochondria of L2C Leydig cells [257]. The expression of Goliath in rat Leydig cells is elevated after hypophysectomy, suggesting that Goliath is regulated by luteinizing hormone. However, the function of this E3 enzyme in mitochondria remains to be determined.

## 4. Conclusions

It is becoming increasingly evident that transmembrane RNF proteins are present in various organelles and play an important role in a number of cellular and organelle functions, such as protein quality control, protein trafficking, cell proliferation and differentiation, apoptosis, immune regulation, signaling and mitochondrial dynamics (see Table 1). In most cases they function as E3 Ub ligases and target membrane proteins for ubiquitination. Defects in their activities have the potential to evoke neuronal and immune abnormalities. In addition to identifying specific substrate proteins, it is important to clarify the mechanisms for their subcellular targeting, substrate recognition and regulation of their activities.

**Table 1:** Summary of known properties of transmembrane RNF proteins.

**Name** *1	**Subcellular localization** *2	**Interacting protein(s)** *3	**Role of interaction**	**Proposed function**	**References**
HRD1 ^b^	ER	Misfolded and native ER proteins (e.g., CFTR)	ERAD substrates	Protein quality control and regulation of protein expression	[74,75,76,77,78,79,80,81,82,83]
		SEL1L, Ubc7, HERP, OS9, XTP3, Derlin proteins	ERAD regulation		
Gp78	ER	Misfolded and native ER proteins (e.g., CFTR)	ERAD substrates		[85,86,89,90,91,92]
		Ubc7	ERAD regulation		
RMA1	ER	Misfolded and native ER proteins (e.g., CFTR)	ERAD substrates		[87,88,92]
		Derlin-1, Ubc6	ERAD regulation		
MARCH6 ^a,b^	ER	Bsep	ERAD substrates	Bile transport	[93,94,95,96]
		DIO2		Regulation of thyroid hormone activity	
		Ubc7	ERAD regulation		
Kf-1	ER	VCP, Derlin-1	ERAD regulation	Regulation of neuronal activity	[97,98,99,100,101]
RFP2	ER	CD3δ, L-type Ca^2+^ channel	ERAD substrates	Protein quality control and regulation of protein expression	[102,103]
		VCP, Derlin-1	ERAD regulation		
		Akt, Mdm2	Substrates	Apoptosis	[145]
ZNRF4 ^b^	ER	Calnexin	ERAD substrate	Regulation of UPR	[21]
TRC8	ER	SREBPs, INSIG-1	ERAD substrates	Cholesterol homeostasis	[107,108]
		SCAP	SREBP-2 trafficking		
RNF170 ^a^	ER	IP_3_R	ERAD substrate	Calcium signaling	[109]
		erlin1, erlin2	Adaptor of IP_3_R		
Dorfin	ER?	CaR	ERAD substrate	Calcium homeostasis	[112]
		VCP	ERAD regulation		
		mutant SOD1	Substrate	Protein quality control	[113,114,115,116]
	Acrosomes	Psmc3	Unknown	Spermiogenesis	[130]
BAR	ER	BI-1	Substrate	UPR signaling	[117]
		Bcl-2, Bcl-xL, caspase-8, caspase-10	Inhibition of apoptotic signaling	Apoptosis	[134,135]
RNF122	ER	CAML	Suppression of E3 activity	Calcium signaling	[122]
Rines ^a,b^	ER	Zic2	Substrate	Unknown	[123]
RNF13 ^a,b^	ER, Golgi, E, Nucleus, Lysosomes	Unknown		Myogenesis, neuronal development and tumorigenesis	[124,125,126,127]
RNF133	ER	Unknown		Spermatogenesis	[129]
RNF144B		p21/WAF, TAp73, ΔNp73	Substrates	Apoptosis	[137,138,139]
	Mitochondria, Cytosol	Activated Bax	Regulation of Bax stability		[143]
RNF182		ATP6V0C	Substrate	Neuronal apoptosis	[144]
RNF152	Lysosomes	Unknown		Lysosome-associated apoptosis	[147]
ZFPL1 ^a,b^	*cis*-Golgi	GM130	Scaffold for the *cis*-Golgi matrix proteins	Assembly of the *cis*-Golgi apparatus	[156]
MARCH1	E, PM, Lysosomes	MHC II, CD86	Substrates	DC maturation and antigen presentation	[164,167,168,169,170,171,173,174,175,176,179]
		CD83	Inhibition of substrate binding		
MARCH8	E, PM	MHC II, CD86	Substrates	T cell development and antigen presentation	[163,164,165,166]
MARCH4	TGN	MHC I, Mult1	Substrates	Immune regulation	[164,182,183]
		CD4	Substrate (K63) *4		
MARCH9	TGN, Lysosomes	MHC I, ICAM-1, Mult1	Substrates	Immune regulation	[164,180,181,182]
GRAIL	E	RhoGDI, CD83, CD81, CD151, CD154, CD3ζ	Substrates	T cell anergy T cell differentiation and activation	[187,188,189,190,191,192]
MARCH2 ^a^	TGN, E, PM	syntaxin-6	Unknown	Endosomal trafficking	[197]
		hDLG1	Substrate	Cell polarity	[19]
MARCH3	TGN, E	syntaxin-6	Unknown	Endosomal trafficking	[198]
MARCH11 ^a^	TGN, E	AP-1	Protein sorting	Spermiogenesis	[199]
MARCH5 ^a,b^	Mitochondria	DRP1, Fis1, MFN1	Substrates	Mitochondrial dynamics	[233,234,235,236]
		MFN2	Unknown		
		mutant SOD1, mutant ataxin-3	Substrates	Protein quality control	[243,244]
		TANK	Substrate (K63) *4	TLR signaling	[247]
MAPL ^b^	Mitochondria Peroxisomes	DRP1	Substrate (sumoylation)	Mitochondrial fission	[20,248,249,250]
		Ubc9	E2 enzyme		
RNF185 ^b^	Mitochondria	BNIP1	Substrate (K63) *4	Mitophagy	[256]
Goliath	Mitochondria	Unknown		Unknown	[257]

*1 ^a^Biochemical evidence for membrane integration; ^b^Biochemical evidence for the presence of the RNF domain in the cytosol.

*2 ER, endoplasmic reticulum; E, endosomes; PM, plasma membrane; TGN, *trans*-Golgi network.

*3 Transmembrane proteins, peripheral membrane proteins, cytosolic proteins and ER luminal proteins are indicated in black, blue, green and purple fonts, respectively.

*4 K63, Lys63-linked ubiquitination.

Based on the published results, several transmembrane E3 ligases fall into three classes: (1) ER-localized E3s, which target substrates for proteasomal degradation to mediate protein quality control, cellular homeostasis and apoptosis; (2) E3s localized in the plasma membrane and endocytic compartments, which promote endocytosis and lysosomal degradation of mainly cell-surface immune regulators; and (3) mitochondrial E3s, which target mitochondria-associated proteins for proteolytic or non-proteolytic ubiquitination to maintain mitochondrial morphology and function. The compartment-specific roles suggest that the subcellular localization of transmembrane E3 ligases is a key determinant of their cellular functions. The localization of some transmembrane RNF proteins is regulated by proteolytic processing [125] and by sorting and scaffold proteins (*i.e.*, AP-1, GM130 and hDLG1) [19,156,199]. Alteration in subcelluler localization of transmembrane E3 ligases is likely to affect their association with substrate proteins, which could be one mechanism controlling their activities and functions.

Although it is likely that transmembrane domains make membrane-bound E3 ligases more accessible to substrate proteins, the recognition mechanism is not precisely known at the molecular level. In some cases, binding proteins facilitate recruiting substrate proteins. For example, the ER luminal lectins OS9 and XTP3 are associated with the luminal side of HRD1 and deliver misfolded luminal proteins to this E3 ligase [76,77,83]. The transmembrane proteins erlin1 and erlin2 act as adaptors between RNF170 and its substrate protein IP_3_R [109]. Substrate recognition is also mediated through protein–protein interaction modules, such as the extacellular/luminal PA domain in GRAIL [33,34] and the cytoplasmic PDZ-binding motif in MARCH2 [19]. Protein–protein interaction also plays an important role in the regulation of E3 activity (*i.e.*, RNF122 and CAML [122]) and substrate binding (*i.e.*, MARCH1 and CD83 [176]). It is necessary to elucidate the functions of the domains and motifs, including the transmembrane domains, for a deeper understanding of the mechanism and requirements of substrate selection and recognition as well as the regulation of action of transmembrane E3 ligases.

A common feature of transmembrane E3 ligases is that they are unstable due to their self-ubiquitination activities, thereby enabling to maintain low ubiquitination activity directed toward substrate proteins at steady state. If this inhibitory mechanism is inhibited in response to certain cellular stimuli, the protein levels of the E3 ligases will increase and ubiquitination of substrate proteins will be promoted. In fact, some deubiquitinating enzymes antagonize self-ubiquitination and stabilize soluble E3 ligases [10]. The diverse functions of E3 ligases are partially conferred by the selectivity of the E2 enzyme that determines the topology of Ub chains. For example, Ubc13 is the only known E2 enzyme that conjugates Lys63-linked Ub chains. Also, Ubc6 and Ubc7 are specifically localized to the ER membrane and participate in ERAD. Therefore, identification of relevant E2 and deubiquitinating enzymes would provide new clues for exploring the regulation of the E3 activities of transmembrane E3 ligases. In addition, determination of the functions of uncharacterized transmembrane RNF proteins would provide novel insights into the regulatory mechanism of Ub-mediated cellular processes, as well as the integrity and functions of cellular and organelle membranes.

## References

[1] Glickman M.H., Ciechanover A. (2002). The ubiquitin-proteasome proteolytic pathway: Destruction for the sake of construction. Physiol. Rev..

[2] Koegl M., Hoppe T., Schlenker S., Ulrich H.D., Mayer T.U., Jentsch S. (1999). A novel ubiquitination factor, E4, is involved in multiubiquitin chain assembly. Cell.

[3] Ikeda F., Dikic I. (2008). Atypical ubiquitin chains: New molecular signals. ‘Protein Modifications: Beyond the Usual Suspects’ review series. EMBO Rep..

[4] Chau V., Tobias J.W., Bachmair A., Marriott D., Ecker D.J., Gonda D.K., Varshavsky A. (1989). A multiubiquitin chain is confined to specific lysine in a targeted short-lived protein. Science.

[5] Thrower J.S., Hoffman L., Rechsteiner M., Pickart C.M. (2000). Recognition of the polyubiquitin proteolytic signal. EMBO J..

[6] Mukhopadhyay D., Riezman H. (2007). Proteasome-independent functions of ubiquitin in endocytosis and signaling. Science.

[7] Bhoj V.G., Chen Z.J. (2009). Ubiquitylation in innate and adaptive immunity. Nature.

[8] Spence J., Sadis S., Haas A.L., Finley D. (1995). A ubiquitin mutant with specific defects in DNA repair and multiubiquitination. Mol. Cell Biol..

[9] Hicke L. (2001). Protein regulation by monoubiquitin. Nat. Rev. Mol. Cell Biol..

[10] Reyes-Turcu F.E., Ventii K.H., Wilkinson K.D. (2009). Regulation and cellular roles of ubiquitin-specific deubiquitinating enzymes. Annu. Rev. Biochem..

[11] Welchman R.L., Gordon C., Mayer R.J. (2005). Ubiquitin and ubiquitin-like proteins as multifunctional signals. Nat. Rev. Mol. Cell Biol..

[12] Ardley H.C., Robinson P.A. (2005). E3 ubiquitin ligases. Essays Biochem..

[13] Rotin D., Kumar S. (2009). Physiological functions of the HECT family of ubiquitin ligases. Nat. Rev. Mol. Cell Biol..

[14] Borden K.L., Boddy M.N., Lally J., O'Reilly N.J., Martin S., Howe K., Solomon E., Freemont P.S. (1995). The solution structure of the RING finger domain from the acute promyelocytic leukaemia proto-oncoprotein PML. EMBO J..

[15] Barlow P.N., Luisi B., Milner A., Elliott M., Everett R. (1994). Structure of the C3HC4 domain by 1H-nuclear magnetic resonance spectroscopy. A new structural class of zinc-finger. J. Mol. Biol..

[16] Dodd R.B., Allen M.D., Brown S.E., Sanderson C.M., Duncan L.M., Lehner P.J., Bycroft M., Read R.J. (2004). Solution structure of the Kaposi's sarcoma-associated herpesvirus K3 N-terminal domain reveals a novel E2-binding C4HC3-type RING domain. J. Biol. Chem..

[17] Deshaies R.J., Joazeiro C.A. (2009). RING domain E3 ubiquitin ligases. Annu. Rev. Biochem..

[18] Ohi M.D., Vander Kooi C.W., Rosenberg J.A., Chazin W.J., Gould K.L. (2003). Structural insights into the U-box, a domain associated with multi-ubiquitination. Nat. Struct. Biol..

[19] Cao Z., Huett A., Kuballa P., Giallourakis C., Xavier R.J. (2008). DLG1 is an anchor for the E3 ligase MARCH2 at sites of cell-cell contact. Cell Signal.

[20] Li W., Bengtson M.H., Ulbrich A., Matsuda A., Reddy V.A., Orth A., Chanda S.K., Batalov S., Joazeiro C.A. (2008). Genome-wide and functional annotation of human E3 ubiquitin ligases identifies MULAN, a mitochondrial E3 that regulates the organelle's dynamics and signaling. PLoS One.

[21] Neutzner A., Neutzner M., Benischke A.S., Ryu S.W., Frank S., Youle R.J., Karbowski M. (2011). A systematic search for endoplasmic reticulum (ER) membrane-associated RING finger proteins identifies Nixin/ZNRF4 as a regulator of calnexin stability and ER homeostasis. J. Biol. Chem..

[22] Thompson J.D., Higgins D.G., Gibson T.J. (1994). CLUSTAL W: Improving the sensitivity of progressive multiple sequence alignment through sequence weighting, position-specific gap penalties and weight matrix choice. Nucleic Acids Res..

[23] Tamura K., Dudley J., Nei M., Kumar S. (2007). MEGA4: Molecular Evolutionary Genetics Analysis (MEGA) software version 4.0. Mol. Biol. Evol..

[24] Meroni G., Diez-Roux G. (2005). TRIM/RBCC, a novel class of ‘single protein RING finger’ E3 ubiquitin ligases. Bioessays.

[25] Massiah M.A., Matts J.A., Short K.M., Simmons B.N., Singireddy S., Yi Z., Cox T.C. (2007). Solution structure of the MID1 B-box2 CHC(D/C)C(2)H(2) zinc-binding domain: Insights into an evolutionarily conserved RING fold. J. Mol. Biol..

[26] Reymond A., Meroni G., Fantozzi A., Merla G., Cairo S., Luzi L., Riganelli D., Zanaria E., Messali S., Cainarca S. (2001). The tripartite motif family identifies cell compartments. EMBO J..

[27] Sardiello M., Cairo S., Fontanella B., Ballabio A., Meroni G. (2008). Genomic analysis of the TRIM family reveals two groups of genes with distinct evolutionary properties. BMC Evol. Biol..

[28] Ensembl Genome Browser (2011). http://www.ensembl.org.

[29] National Center for Biotechnology Information (2011). http://www.ncbi.nlm.nih.gov.

[30] Jin X., Cheng H., Chen J., Zhu D. (2011). RNF13: An emerging RING finger ubiquitin ligase important in cell proliferation. FEBS J..

[31] Whiting C.C., Su L.L., Lin J.T., Fathman C.G. (2011). GRAIL: A unique mediator of CD4 T-lymphocyte unresponsiveness. FEBS J..

[32] Mahon P., Bateman A. (2000). The PA domain: A protease-associated domain. Protein Sci..

[33] Su L.L., Iwai H., Lin J.T., Fathman C.G. (2009). The transmembrane E3 ligase GRAIL ubiquitinates and degrades CD83 on CD4 T cells. J. Immunol..

[34] Lineberry N.B., Su L.L., Lin J.T., Coffey G.P., Seroogy C.M., Fathman C.G. (2008). Cutting edge: The transmembrane E3 ligase GRAIL ubiquitinates the costimulatory molecule CD40 ligand during the induction of T cell anergy. J. Immunol..

[35] Bocock J.P., Carmicle S., Sircar M., Erickson A.H. (2011). Trafficking and proteolytic processing of RNF13, a model PA-TM-RING family endosomal membrane ubiquitin ligase. FEBS J..

[36] Nathan J.A., Lehner P.J. (2009). The trafficking and regulation of membrane receptors by the RING-CH ubiquitin E3 ligases. Exp. Cell Res..

[37] Iyengar P.V., Hirota T., Hirose S., Nakamura N. (2011). Membrane-associated RING-CH 10 (MARCH10 protein) is a microtubule-associated E3 ubiquitin ligase of the spermatid flagella. J. Biol. Chem..

[38] Nathan J.A., Sengupta S., Wood S.A., Admon A., Markson G., Sanderson C., Lehner P.J. (2008). The ubiquitin E3 ligase MARCH7 is differentially regulated by the deubiquitylating enzymes USP7 and USP9X. Traffic.

[39] Bezprozvanny I., Maximov A. (2001). Classification of PDZ domains. FEBS Lett..

[40] Kay B.K., Williamson M.P., Sudol M. (2000). The importance of being proline: The interaction of proline-rich motifs in signaling proteins with their cognate domains. FASEB J..

[41] Eisenhaber B., Chumak N., Eisenhaber F., Hauser M.T. (2007). The ring between ring fingers (RBR) protein family. Genome Biol..

[42] Capili A.D., Edghill E.L., Wu K., Borden K.L. (2004). Structure of the C-terminal RING finger from a RING-IBR-RING/TRIAD motif reveals a novel zinc-binding domain distinct from a RING. J. Mol. Biol..

[43] Stevens T.J., Arkin I.T. (2000). Do more complex organisms have a greater proportion of membrane proteins in their genomes?. Proteins.

[44] Lander E.S., Linton L.M., Birren B., Nusbaum C., Zody M.C., Baldwin J., Devon K., Dewar K., Doyle M., FitzHugh W. (2001). Initial sequencing and analysis of the human genome. Nature.

[45] Diehn M., Bhattacharya R., Botstein D., Brown P.O. (2006). Genome-scale identification of membrane-associated human mRNAs. PLoS Genet..

[46] Rapoport T.A. (2007). Protein translocation across the eukaryotic endoplasmic reticulum and bacterial plasma membranes. Nature.

[47] Sitia R., Braakman I. (2003). Quality control in the endoplasmic reticulum protein factory. Nature.

[48] Malhotra J.D., Kaufman R.J. (2007). The endoplasmic reticulum and the unfolded protein response. Semin. Cell Dev. Biol..

[49] Vembar S.S., Brodsky J.L. (2008). One step at a time: Endoplasmic reticulum-associated degradation. Nat. Rev. Mol. Cell Biol..

[50] Wojcikiewicz R.J. (2004). Regulated ubiquitination of proteins in GPCR-initiated signaling pathways. Trends Pharmacol. Sci..

[51] Espenshade P.J., Hughes A.L. (2007). Regulation of sterol synthesis in eukaryotes. Annu. Rev. Genet..

[52] Shamu C.E., Flierman D., Ploegh H.L., Rapoport T.A., Chau V. (2001). Polyubiquitination is required for US11-dependent movement of MHC class I heavy chain from endoplasmic reticulum into cytosol. Mol. Biol. Cell.

[53] De Virgilio M., Weninger H., Ivessa N.E. (1998). Ubiquitination is required for the retro-translocation of a short-lived luminal endoplasmic reticulum glycoprotein to the cytosol for degradation by the proteasome. J. Biol. Chem..

[54] Plemper R.K., Bohmler S., Bordallo J., Sommer T., Wolf D.H. (1997). Mutant analysis links the translocon and BiP to retrograde protein transport for ER degradation. Nature.

[55] Meusser B., Hirsch C., Jarosch E., Sommer T. (2005). ERAD: The long road to destruction. Nat. Cell Biol..

[56] Wiertz E.J., Tortorella D., Bogyo M., Yu J., Mothes W., Jones T.R., Rapoport T.A., Ploegh H.L. (1996). Sec61-mediated transfer of a membrane protein from the endoplasmic reticulum to the proteasome for destruction. Nature.

[57] Ye Y., Meyer H.H., Rapoport T.A. (2001). The AAA ATPase Cdc48/p97 and its partners transport proteins from the ER into the cytosol. Nature.

[58] Jarosch E., Taxis C., Volkwein C., Bordallo J., Finley D., Wolf D.H., Sommer T. (2002). Protein dislocation from the ER requires polyubiquitination and the AAA-ATPase Cdc48. Nat. Cell Biol..

[59] Braun S., Matuschewski K., Rape M., Thoms S., Jentsch S. (2002). Role of the ubiquitin-selective CDC48(UFD1/NPL4) chaperone (segregase) in ERAD of OLE1 and other substrates. EMBO J..

[60] Bays N.W., Wilhovsky S.K., Goradia A., Hodgkiss-Harlow K., Hampton R.Y. (2001). HRD4/NPL4 is required for the proteasomal processing of ubiquitinated ER proteins. Mol. Biol. Cell.

[61] Lilley B.N., Ploegh H.L. (2005). Multiprotein complexes that link dislocation, ubiquitination, and extraction of misfolded proteins from the endoplasmic reticulum membrane. Proc. Natl. Acad. Sci. USA.

[62] Ye Y., Shibata Y., Kikkert M., van Voorden S., Wiertz E., Rapoport T.A. (2005). Recruitment of the p97 ATPase and ubiquitin ligases to the site of retrotranslocation at the endoplasmic reticulum membrane. Proc. Natl. Acad. Sci. USA.

[63] Vashist S., Ng D.T. (2004). Misfolded proteins are sorted by a sequential checkpoint mechanism of ER quality control. J. Cell Biol..

[64] Gauss R., Jarosch E., Sommer T., Hirsch C. (2006). A complex of Yos9p and the HRD ligase integrates endoplasmic reticulum quality control into the degradation machinery. Nat. Cell Biol..

[65] Denic V., Quan E.M., Weissman J.S. (2006). A luminal surveillance complex that selects misfolded glycoproteins for ER-associated degradation. Cell.

[66] Carvalho P., Goder V., Rapoport T.A. (2006). Distinct ubiquitin-ligase complexes define convergent pathways for the degradation of ER proteins. Cell.

[67] Biederer T., Volkwein C., Sommer T. (1997). Role of Cue1p in ubiquitination and degradation at the ER surface. Science.

[68] Deak P.M., Wolf D.H. (2001). Membrane topology and function of Der3/Hrd1p as a ubiquitin-protein ligase (E3) involved in endoplasmic reticulum degradation. J. Biol. Chem..

[69] Bays N.W., Gardner R.G., Seelig L.P., Joazeiro C.A., Hampton R.Y. (2001). Hrd1p/Der3p is a membrane-anchored ubiquitin ligase required for ER-associated degradation. Nat. Cell Biol..

[70] Swanson R., Locher M., Hochstrasser M. (2001). A conserved ubiquitin ligase of the nuclear envelope/endoplasmic reticulum that functions in both ER-associated and Matalpha2 repressor degradation. Genes Dev..

[71] Mehnert M., Sommer T., Jarosch E. (2010). ERAD ubiquitin ligases: Multifunctional tools for protein quality control and waste disposal in the endoplasmic reticulum. Bioessays.

[72] Feldman M., van der Goot F.G. (2009). Novel ubiquitin-dependent quality control in the endoplasmic reticulum. Trends Cell Biol..

[73] Hirsch C., Gauss R., Horn S.C., Neuber O., Sommer T. (2009). The ubiquitylation machinery of the endoplasmic reticulum. Nature.

[74] Nadav E., Shmueli A., Barr H., Gonen H., Ciechanover A., Reiss Y. (2003). A novel mammalian endoplasmic reticulum ubiquitin ligase homologous to the yeast Hrd1. Biochem. Biophys. Res. Commun..

[75] Kikkert M., Doolman R., Dai M., Avner R., Hassink G., van Voorden S., Thanedar S., Roitelman J., Chau V., Wiertz E. (2004). Human HRD1 is an E3 ubiquitin ligase involved in degradation of proteins from the endoplasmic reticulum. J. Biol. Chem..

[76] Christianson J.C., Shaler T.A., Tyler R.E., Kopito R.R. (2008). OS-9 and GRP94 deliver mutant alpha1-antitrypsin to the Hrd1-SEL1L ubiquitin ligase complex for ERAD. Nat. Cell Biol..

[77] Bernasconi R., Pertel T., Luban J., Molinari M. (2008). A dual task for the Xbp1-responsive OS-9 variants in the mammalian endoplasmic reticulum: Inhibiting secretion of misfolded protein conformers and enhancing their disposal. J. Biol. Chem..

[78] Ye Y., Shibata Y., Yun C., Ron D., Rapoport T.A. (2004). A membrane protein complex mediates retro-translocation from the ER lumen into the cytosol. Nature.

[79] Lilley B.N., Ploegh H.L. (2004). A membrane protein required for dislocation of misfolded proteins from the ER. Nature.

[80] Schulze A., Standera S., Buerger E., Kikkert M., van Voorden S., Wiertz E., Koning F., Kloetzel P.M., Seeger M. (2005). The ubiquitin-domain protein HERP forms a complex with components of the endoplasmic reticulum associated degradation pathway. J. Mol. Biol..

[81] Kokame K., Agarwala K.L., Kato H., Miyata T. (2000). Herp, a new ubiquitin-like membrane protein induced by endoplasmic reticulum stress. J. Biol. Chem..

[82] Mueller B., Lilley B.N., Ploegh H.L. (2006). SEL1L, the homologue of yeast Hrd3p, is involved in protein dislocation from the mammalian ER. J. Cell Biol..

[83] Hosokawa N., Wada I., Nagasawa K., Moriyama T., Okawa K., Nagata K. (2008). Human XTP3-B forms an endoplasmic reticulum quality control scaffold with the HRD1-SEL1L ubiquitin ligase complex and BiP. J. Biol. Chem..

[84] Oda Y., Okada T., Yoshida H., Kaufman R.J., Nagata K., Mori K. (2006). Derlin-2 and Derlin-3 are regulated by the mammalian unfolded protein response and are required for ER-associated degradation. J. Cell Biol..

[85] Chen B., Mariano J., Tsai Y.C., Chan A.H., Cohen M., Weissman A.M. (2006). The activity of a human endoplasmic reticulum-associated degradation E3, gp78, requires its Cue domain, RING finger, and an E2-binding site. Proc. Natl. Acad. Sci. USA.

[86] Fang S., Ferrone M., Yang C., Jensen J.P., Tiwari S., Weissman A.M. (2001). The tumor autocrine motility factor receptor, gp78, is a ubiquitin protein ligase implicated in degradation from the endoplasmic reticulum. Proc. Natl. Acad. Sci. USA.

[87] Younger J.M., Chen L., Ren H.Y., Rosser M.F., Turnbull E.L., Fan C.Y., Patterson C., Cyr D.M. (2006). Sequential quality-control checkpoints triage misfolded cystic fibrosis transmembrane conductance regulator. Cell.

[88] Matsuda N., Suzuki T., Tanaka K., Nakano A. (2001). Rma1, a novel type of RING finger protein conserved from Arabidopsis to human, is a membrane-bound ubiquitin ligase. J. Cell Sci..

[89] Tsai Y.C., Mendoza A., Mariano J.M., Zhou M., Kostova Z., Chen B., Veenstra T., Hewitt S.M., Helman L.J., Khanna C., Weissman A.M. (2007). The ubiquitin ligase gp78 promotes sarcoma metastasis by targeting KAI1 for degradation. Nat. Med..

[90] Song B.L., Sever N., DeBose-Boyd R.A. (2005). Gp78, a membrane-anchored ubiquitin ligase, associates with Insig-1 and couples sterol-regulated ubiquitination to degradation of HMG CoA reductase. Mol. Cell.

[91] Liang J.S., Kim T., Fang S., Yamaguchi J., Weissman A.M., Fisher E.A., Ginsberg H.N. (2003). Overexpression of the tumor autocrine motility factor receptor Gp78, a ubiquitin protein ligase, results in increased ubiquitinylation and decreased secretion of apolipoprotein B100 in HepG2 cells. J. Biol. Chem..

[92] Morito D., Hirao K., Oda Y., Hosokawa N., Tokunaga F., Cyr D.M., Tanaka K., Iwai K., Nagata K. (2008). Gp78 cooperates with RMA1 in endoplasmic reticulum-associated degradation of CFTRDeltaF508. Mol. Biol. Cell.

[93] Kreft S.G., Wang L., Hochstrasser M. (2006). Membrane topology of the yeast endoplasmic reticulum-localized ubiquitin ligase Doa10 and comparison with its human ortholog TEB4 (MARCH-VI). J. Biol. Chem..

[94] Hassink G., Kikkert M., van Voorden S., Lee S.J., Spaapen R., van Laar T., Coleman C.S., Bartee E., Fruh K., Chau V. (2005). TEB4 is a C4HC3 RING finger-containing ubiquitin ligase of the endoplasmic reticulum. Biochem. J..

[95] Zavacki A.M., Arrojo E.D.R., Freitas B.C., Chung M., Harney J.W., Egri P., Wittmann G., Fekete C., Gereben B., Bianco A.C. (2009). The E3 ubiquitin ligase TEB4 mediates degradation of type 2 iodothyronine deiodinase. Mol. Cell Biol..

[96] Wang L., Dong H., Soroka C.J., Wei N., Boyer J.L., Hochstrasser M. (2008). Degradation of the bile salt export pump at endoplasmic reticulum in progressive familial intrahepatic cholestasis type II. Hepatology.

[97] Yasojima K., Tsujimura A., Mizuno T., Shigeyoshi Y., Inazawa J., Kikuno R., Kuma K., Ohkubo K., Hosokawa Y., Ibata Y. (1997). Cloning of human and mouse cDNAs encoding novel zinc finger proteins expressed in cerebellum and hippocampus. Biochem. Biophys. Res. Commun..

[98] Maruyama Y., Yamada M., Takahashi K. (2008). Ubiquitin ligase Kf-1 is involved in the endoplasmic reticulum-associated degradation pathway. Biochem. Biophys. Res. Commun..

[99] Nishioka G., Yamada M., Kudo K., Takahashi K., Kiuchi Y., Higuchi T., Momose K., Kamijima K. (2003). Induction of kf-1 after repeated electroconvulsive treatment and chronic antidepressant treatment in rat frontal cortex and hippocampus. J. Neural Transm..

[100] Yamada M., Yamazaki S., Takahashi K., Nishioka G., Kudo K., Ozawa H., Yamada S., Kiuchi Y., Kamijima K., Higuchi T. (2000). Identification of a novel gene with RING-H2 finger motif induced after chronic antidepressant treatment in rat brain. Biochem. Biophys. Res. Commun..

[101] Hashimoto-Gotoh T., Iwabe N., Tsujimura A., Nakagawa M., Marunaka Y. (2011). KF-1 ubiquitin ligase: Anxiety suppressor model. Cell Biochem. Biophys..

[102] Altier C., Garcia-Caballero A., Simms B., You H., Chen L., Walcher J., Tedford H.W., Hermosilla T., Zamponi G.W. (2011). The Cavbeta subunit prevents RFP2-mediated ubiquitination and proteasomal degradation of L-type channels. Nat. Neurosci..

[103] Lerner M., Corcoran M., Cepeda D., Nielsen M.L., Zubarev R., Ponten F., Uhlen M., Hober S., Grander D., Sangfelt O. (2007). The RBCC gene RFP2 (Leu5) encodes a novel transmembrane E3 ubiquitin ligase involved in ERAD. Mol. Biol. Cell.

[104] Goldstein J.L., DeBose-Boyd R.A., Brown M.S. (2006). Protein sensors for membrane sterols. Cell.

[105] Gemmill R.M., Bemis L.T., Lee J.P., Sozen M.A., Baron A., Zeng C., Erickson P.F., Hooper J.E., Drabkin H.A. (2002). The TRC8 hereditary kidney cancer gene suppresses growth and functions with VHL in a common pathway. Oncogene.

[106] Gemmill R.M., West J.D., Boldog F., Tanaka N., Robinson L.J., Smith D.I., Li F., Drabkin H.A. (1998). The hereditary renal cell carcinoma 3;8 translocation fuses FHIT to a patched-related gene, TRC8. Proc. Natl. Acad. Sci. USA.

[107] Irisawa M., Inoue J., Ozawa N., Mori K., Sato R. (2009). The sterol-sensing endoplasmic reticulum (ER) membrane protein TRC8 hampers ER to Golgi transport of sterol regulatory element-binding protein-2 (SREBP-2)/SREBP cleavage-activated protein and reduces SREBP-2 cleavage. J. Biol. Chem..

[108] Lee J.P., Brauweiler A., Rudolph M., Hooper J.E., Drabkin H.A., Gemmill R.M. (2010). The TRC8 ubiquitin ligase is sterol regulated and interacts with lipid and protein biosynthetic pathways. Mol. Cancer Res..

[109] Lu J.P., Wang Y., Sliter D.A., Pearce M.M., Wojcikiewicz R.J. (2011). RNF170 protein, an endoplasmic reticulum membrane ubiquitin ligase, mediates inositol 1,4,5-trisphosphate receptor ubiquitination and degradation. J. Biol. Chem..

[110] Valdmanis P.N., Dupre N., Lachance M., Stochmanski S.J., Belzil V.V., Dion P.A., Thiffault I., Brais B., Weston L., Saint-Amant L., Samuels M.E., Rouleau G.A. (2011). A mutation in the RNF170 gene causes autosomal dominant sensory ataxia. Brain.

[111] Brown E.M., MacLeod R.J. (2001). Extracellular calcium sensing and extracellular calcium signaling. Physiol. Rev..

[112] Huang Y., Niwa J., Sobue G., Breitwieser G.E. (2006). Calcium-sensing receptor ubiquitination and degradation mediated by the E3 ubiquitin ligase dorfin. J. Biol. Chem..

[113] Niwa J., Ishigaki S., Hishikawa N., Yamamoto M., Doyu M., Murata S., Tanaka K., Taniguchi N., Sobue G. (2002). Dorfin ubiquitylates mutant SOD1 and prevents mutant SOD1-mediated neurotoxicity. J. Biol. Chem..

[114] Sone J., Niwa J., Kawai K., Ishigaki S., Yamada S., Adachi H., Katsuno M., Tanaka F., Doyu M., Sobue G. (2010). Dorfin ameliorates phenotypes in a transgenic mouse model of amyotrophic lateral sclerosis. J. Neurosci. Res..

[115] Niwa J., Yamada S., Ishigaki S., Sone J., Takahashi M., Katsuno M., Tanaka F., Doyu M., Sobue G. (2007). Disulfide bond mediates aggregation, toxicity, and ubiquitylation of familial amyotrophic lateral sclerosis-linked mutant SOD1. J. Biol. Chem..

[116] Takeuchi H., Niwa J., Hishikawa N., Ishigaki S., Tanaka F., Doyu M., Sobue G. (2004). Dorfin prevents cell death by reducing mitochondrial localizing mutant superoxide dismutase 1 in a neuronal cell model of familial amyotrophic lateral sclerosis. J. Neurochem..

[117] Rong J., Chen L., Toth J.I., Tcherpakov M., Petroski M.D., Reed J.C. (2011). Bifunctional apoptosis regulator (BAR), an endoplasmic reticulum (ER)-associated E3 ubiquitin ligase, modulates BI-1 protein stability and function in ER Stress. J. Biol. Chem..

[118] Cox J.S., Shamu C.E., Walter P. (1993). Transcriptional induction of genes encoding endoplasmic reticulum resident proteins requires a transmembrane protein kinase. Cell.

[119] Yoshida H., Matsui T., Yamamoto A., Okada T., Mori K. (2001). XBP1 mRNA is induced by ATF6 and spliced by IRE1 in response to ER stress to produce a highly active transcription factor. Cell.

[120] Calfon M., Zeng H., Urano F., Till J.H., Hubbard S.R., Harding H.P., Clark S.G., Ron D. (2002). IRE1 couples endoplasmic reticulum load to secretory capacity by processing the XBP-1 mRNA. Nature.

[121] Urano F., Wang X., Bertolotti A., Zhang Y., Chung P., Harding H.P., Ron D. (2000). Coupling of stress in the ER to activation of JNK protein kinases by transmembrane protein kinase IRE1. Science.

[122] Peng Z., Shi T., Ma D. (2010). RNF122: A novel ubiquitin ligase associated with calcium-modulating cyclophilin ligand. BMC Cell Biol..

[123] Ogawa M., Mizugishi K., Ishiguro A., Koyabu Y., Imai Y., Takahashi R., Mikoshiba K., Aruga J. (2008). Rines/RNF180, a novel RING finger gene-encoded product, is a membrane-bound ubiquitin ligase. Genes Cells.

[124] Zhang Q., Wang K., Zhang Y., Meng J., Yu F., Chen Y., Zhu D. (2010). The myostatin-induced E3 ubiquitin ligase RNF13 negatively regulates the proliferation of chicken myoblasts. FEBS J..

[125] Bocock J.P., Carmicle S., Chhotani S., Ruffolo M.R., Chu H., Erickson A.H. (2009). The PA-TM-RING protein RING finger protein 13 is an endosomal integral membrane E3 ubiquitin ligase whose RING finger domain is released to the cytoplasm by proteolysis. FEBS J..

[126] Zhang Q., Meng Y., Zhang L., Chen J., Zhu D. (2009). RNF13: A novel RING-type ubiquitin ligase over-expressed in pancreatic cancer. Cell Res..

[127] Bocock J.P., Carmicle S., Madamba E., Erickson A.H. (2010). Nuclear targeting of an endosomal E3 ubiquitin ligase. Traffic.

[128] Schultz N., Hamra F.K., Garbers D.L. (2003). A multitude of genes expressed solely in meiotic or postmeiotic spermatogenic cells offers a myriad of contraceptive targets. Proc. Natl. Acad. Sci. USA.

[129] Nian H., Zhang W., Shi H., Zhao Q., Xie Q., Liao S., Zhang Y., Zhang Z., Wang C., Han C. (2008). Mouse RING finger protein Rnf133 is a testis-specific endoplasmic reticulum-associated E3 ubiquitin ligase. Cell Res..

[130] Rivkin E., Kierszenbaum A.L., Gil M., Tres L.L. (2009). Rnf19a, a ubiquitin protein ligase, and Psmc3, a component of the 26S proteasome, tether to the acrosome membranes and the head-tail coupling apparatus during rat spermatid development. Dev. Dyn..

[131] French L.E., Tschopp J. (2003). Protein-based therapeutic approaches targeting death receptors. Cell Death Differ..

[132] Garrido C., Galluzzi L., Brunet M., Puig P.E., Didelot C., Kroemer G. (2006). Mechanisms of cytochrome c release from mitochondria. Cell Death Differ..

[133] Gross A., McDonnell J.M., Korsmeyer S.J. (1999). BCL-2 family members and the mitochondria in apoptosis. Genes Dev..

[134] Zhang H., Xu Q., Krajewski S., Krajewska M., Xie Z., Fuess S., Kitada S., Pawlowski K., Godzik A., Reed J.C. (2000). BAR: An apoptosis regulator at the intersection of caspases and Bcl-2 family proteins. Proc. Natl. Acad. Sci. USA.

[135] Roth W., Kermer P., Krajewska M., Welsh K., Davis S., Krajewski S., Reed J.C. (2003). Bifunctional apoptosis inhibitor (BAR) protects neurons from diverse cell death pathways. Cell Death Differ..

[136] Oren M. (2003). Decision making by p53: Life, death and cancer. Cell Death Differ..

[137] Ng C.C., Arakawa H., Fukuda S., Kondoh H., Nakamura Y. (2003). p53RFP, a p53-inducible RING-finger protein, regulates the stability of p21WAF1. Oncogene.

[138] Huang J., Xu L.G., Liu T., Zhai Z., Shu H.B. (2006). The p53-inducible E3 ubiquitin ligase p53RFP induces p53-dependent apoptosis. FEBS Lett..

[139] Sayan B.S., Yang A.L., Conforti F., Tucci P., Piro M.C., Browne G.J., Agostini M., Bernardini S., Knight R.A., Mak T.W. (2010). Differential control of TAp73 and DeltaNp73 protein stability by the ring finger ubiquitin ligase PIR2. Proc. Natl. Acad. Sci. USA.

[140] Ishimoto O., Kawahara C., Enjo K., Obinata M., Nukiwa T., Ikawa S. (2002). Possible oncogenic potential of DeltaNp73: A newly identified isoform of human p73. Cancer Res..

[141] Jost C.A., Marin M.C., Kaelin W.G. (1997). p73 is a simian [correction of human] p53-related protein that can induce apoptosis. Nature.

[142] Maisse C., Munarriz E., Barcaroli D., Melino G., De Laurenzi V. (2004). DNA damage induces the rapid and selective degradation of the DeltaNp73 isoform, allowing apoptosis to occur. Cell Death Differ..

[143] Benard G., Neutzner A., Peng G., Wang C., Livak F., Youle R.J., Karbowski M. (2010). IBRDC2, an IBR-type E3 ubiquitin ligase, is a regulatory factor for Bax and apoptosis activation. EMBO J..

[144] Liu Q.Y., Lei J.X., Sikorska M., Liu R. (2008). A novel brain-enriched E3 ubiquitin ligase RNF182 is up regulated in the brains of Alzheimer's patients and targets ATP6V0C for degradation. Mol. Neurodegener..

[145] Joo H.M., Kim J.Y., Jeong J.B., Seong K.M., Nam S.Y., Yang K.H., Kim C.S., Kim H.S., Jeong M., An S. (2011). Ret finger protein 2 enhances ionizing radiation-induced apoptosis via degradation of AKT and MDM2. Eur J. Cell Biol..

[146] Guicciardi M.E., Leist M., Gores G.J. (2004). Lysosomes in cell death. Oncogene.

[147] Zhang S., Wu W., Wu Y., Zheng J., Suo T., Tang H., Tang J. (2010). RNF152, a novel lysosome localized E3 ligase with pro-apoptotic activities. Protein Cell.

[148] Wilson C., Ragnini-Wilson A. (2010). Conserved molecular mechanisms underlying homeostasis of the Golgi complex. Int. J. Cell Biol..

[149] Sciaky N., Presley J., Smith C., Zaal K.J., Cole N., Moreira J.E., Terasaki M., Siggia E., Lippincott-Schwartz J. (1997). Golgi tubule traffic and the effects of brefeldin A visualized in living cells. J. Cell Biol..

[150] Ramirez I.B., Lowe M. (2009). Golgins and GRASPs: Holding the Golgi together. Semin. Cell Dev. Biol..

[151] Barr F.A., Nakamura N., Warren G. (1998). Mapping the interaction between GRASP65 and GM130, components of a protein complex involved in the stacking of Golgi cisternae. EMBO J..

[152] Barr F.A., Puype M., Vandekerckhove J., Warren G. (1997). GRASP65, a protein involved in the stacking of Golgi cisternae. Cell.

[153] Nakamura N., Lowe M., Levine T.P., Rabouille C., Warren G. (1997). The vesicle docking protein p115 binds GM130, a *cis*-Golgi matrix protein, in a mitotically regulated manner. Cell.

[154] Lesa G.M., Seemann J., Shorter J., Vandekerckhove J., Warren G. (2000). The amino-terminal domain of the golgi protein giantin interacts directly with the vesicle-tethering protein p115. J. Biol. Chem..

[155] Diao A., Frost L., Morohashi Y., Lowe M. (2008). Coordination of golgin tethering and SNARE assembly: GM130 binds syntaxin 5 in a p115-regulated manner. J. Biol. Chem..

[156] Chiu C.F., Ghanekar Y., Frost L., Diao A., Morrison D., McKenzie E., Lowe M. (2008). ZFPL1, a novel ring finger protein required for *cis*-Golgi integrity and efficient ER-to-Golgi transport. EMBO J..

[157] Dupre S., Urban-Grimal D., Haguenauer-Tsapis R. (2004). Ubiquitin and endocytic internalization in yeast and animal cells. Biochim. Biophys. Acta.

[158] Piper R.C., Luzio J.P. (2007). Ubiquitin-dependent sorting of integral membrane proteins for degradation in lysosomes. Curr. Opin. Cell Biol..

[159] Ren X., Hurley J.H. (2010). VHS domains of ESCRT-0 cooperate in high-avidity binding to polyubiquitinated cargo. EMBO J..

[160] Katzmann D.J., Babst M., Emr S.D. (2001). Ubiquitin-dependent sorting into the multivesicular body pathway requires the function of a conserved endosomal protein sorting complex, ESCRT-I. Cell.

[161] Raiborg C., Stenmark H. (2009). The ESCRT machinery in endosomal sorting of ubiquitylated membrane proteins. Nature.

[162] Ishido S., Matsuki Y., Goto E., Kajikawa M., Ohmura-Hoshino M. (2010). MARCH-I: A new regulator of dendritic cell function. Mol. Cells.

[163] Goto E., Ishido S., Sato Y., Ohgimoto S., Ohgimoto K., Nagano-Fujii M., Hotta H. (2003). c-MIR, a human E3 ubiquitin ligase, is a functional homolog of herpesvirus proteins MIR1 and MIR2 and has similar activity. J. Biol. Chem..

[164] Bartee E., Mansouri M., Hovey Nerenberg B.T., Gouveia K., Fruh K. (2004). Downregulation of major histocompatibility complex class I by human ubiquitin ligases related to viral immune evasion proteins. J. Virol..

[165] Ohmura-Hoshino M., Matsuki Y., Aoki M., Goto E., Mito M., Uematsu M., Kakiuchi T., Hotta H., Ishido S. (2006). Inhibition of MHC class II expression and immune responses by c-MIR. J. Immunol..

[166] Goto E., Mito-Yoshida M., Uematsu M., Aoki M., Matsuki Y., Ohmura-Hoshino M., Hotta H., Miyagishi M., Ishido S. (2008). An excellent monitoring system for surface ubiquitination-induced internalization in mammals. PLoS One.

[167] Matsuki Y., Ohmura-Hoshino M., Goto E., Aoki M., Mito-Yoshida M., Uematsu M., Hasegawa T., Koseki H., Ohara O., Nakayama M. (2007). Novel regulation of MHC class II function in B cells. EMBO J..

[168] Baravalle G., Park H., McSweeney M., Ohmura-Hoshino M., Matsuki Y., Ishido S., Shin J.S. (2011). Ubiquitination of CD86 is a key mechanism in regulating antigen presentation by dendritic cells. J. Immunol..

[169] Corcoran K., Jabbour M., Bhagwandin C., Deymier M.J., Theisen D.L., Lybarger L. (2011). Ubiquitin-mediated regulation of CD86 expression by membrane-associated RING-CH1 (MARCH1). J. Biol. Chem..

[170] Ohmura-Hoshino M., Matsuki Y., Mito-Yoshida M., Goto E., Aoki-Kawasumi M., Nakayama M., Ohara O., Ishido S. (2009). Cutting edge: Requirement of MARCH-I-mediated MHC II ubiquitination for the maintenance of conventional dendritic cells. J. Immunol..

[171] Walseng E., Furuta K., Bosch B., Weih K.A., Matsuki Y., Bakke O., Ishido S., Roche P.A. (2010). Ubiquitination regulates MHC class II-peptide complex retention and degradation in dendritic cells. Proc. Natl. Acad. Sci. USA.

[172] Walseng E., Furuta K., Goldszmid R.S., Weih K.A., Sher A., Roche P.A. (2010). Dendritic cell activation prevents MHC class II ubiquitination and promotes MHC class II survival regardless of the activation stimulus. J. Biol. Chem..

[173] Van Niel G., Wubbolts R., Ten Broeke T., Buschow S.I., Ossendorp F.A., Melief C.J., Raposo G., van Balkom B.W., Stoorvogel W. (2006). Dendritic cells regulate exposure of MHC class II at their plasma membrane by oligoubiquitination. Immunity.

[174] Jabbour M., Campbell E.M., Fares H., Lybarger L. (2009). Discrete domains of MARCH1 mediate its localization, functional interactions, and posttranscriptional control of expression. J. Immunol..

[175] De Gassart A., Camosseto V., Thibodeau J., Ceppi M., Catalan N., Pierre P., Gatti E. (2008). MHC class II stabilization at the surface of human dendritic cells is the result of maturation-dependent MARCH I down-regulation. Proc. Natl. Acad. Sci. USA.

[176] Tze L.E., Horikawa K., Domaschenz H., Howard D.R., Roots C.M., Rigby R.J., Way D.A., Ohmura-Hoshino M., Ishido S., Andoniou C.E. (2011). CD83 increases MHC II and CD86 on dendritic cells by opposing IL-10-driven MARCH1-mediated ubiquitination and degradation. J. Exp. Med..

[177] Pletinckx K., Dohler A., Pavlovic V., Lutz M.B. (2011). Role of dendritic cell maturity/costimulation for generation, homeostasis and suppressive activity of regulatory T cells. Front. Immunol..

[178] Corinti S., Albanesi C., la Sala A., Pastore S., Girolomoni G. (2001). Regulatory activity of autocrine IL-10 on dendritic cell functions. J. Immunol..

[179] Thibodeau J., Bourgeois-Daigneault M.C., Huppe G., Tremblay J., Aumont A., Houde M., Bartee E., Brunet A., Gauvreau M.E., de Gassart A. (2008). Interleukin-10-induced MARCH1 mediates intracellular sequestration of MHC class II in monocytes. Eur. J. Immunol..

[180] Hor S., Ziv T., Admon A., Lehner P.J. (2009). Stable isotope labeling by amino acids in cell culture and differential plasma membrane proteome quantitation identify new substrates for the MARCH9 transmembrane E3 ligase. Mol. Cell Proteomics.

[181] Hoer S., Smith L., Lehner P.J. (2007). MARCH-IX mediates ubiquitination and downregulation of ICAM-1. FEBS Lett..

[182] Nice T.J., Deng W., Coscoy L., Raulet D.H. (2010). Stress-regulated targeting of the NKG2D ligand Mult1 by a membrane-associated RING-CH family E3 ligase. J. Immunol..

[183] Barriere H., Nemes C., Du K., Lukacs G.L. (2007). Plasticity of polyubiquitin recognition as lysosomal targeting signals by the endosomal sorting machinery. Mol. Biol. Cell.

[184] Eyster C.A., Cole N.B., Petersen S., Viswanathan K., Fruh K., Donaldson J.G. (2011). MARCH ubiquitin ligases alter the itinerary of clathrin-independent cargo from recycling to degradation. Mol. Biol. Cell.

[185] Appleman L.J., Boussiotis V.A. (2003). T cell anergy and costimulation. Immunol. Rev..

[186] Schwartz R.H. (2003). T cell anergy. Annu. Rev. Immunol..

[187] Anandasabapathy N., Ford G.S., Bloom D., Holness C., Paragas V., Seroogy C., Skrenta H., Hollenhorst M., Fathman C.G., Soares L. (2003). GRAIL: An E3 ubiquitin ligase that inhibits cytokine gene transcription is expressed in anergic CD4+ T cells. Immunity.

[188] Heissmeyer V., Macian F., Im S.H., Varma R., Feske S., Venuprasad K., Gu H., Liu Y.C., Dustin M.L., Rao A. (2004). Calcineurin imposes T cell unresponsiveness through targeted proteolysis of signaling proteins. Nat. Immunol..

[189] Seroogy C.M., Soares L., Ranheim E.A., Su L., Holness C., Bloom D., Fathman C.G. (2004). The gene related to anergy in lymphocytes, an E3 ubiquitin ligase, is necessary for anergy induction in CD4 T cells. J. Immunol..

[190] Lineberry N., Su L., Soares L., Fathman C.G. (2008). The single subunit transmembrane E3 ligase gene related to anergy in lymphocytes (GRAIL) captures and then ubiquitinates transmembrane proteins across the cell membrane. J. Biol. Chem..

[191] Su L., Lineberry N., Huh Y., Soares L., Fathman C.G. (2006). A novel E3 ubiquitin ligase substrate screen identifies Rho guanine dissociation inhibitor as a substrate of gene related to anergy in lymphocytes. J. Immunol..

[192] Kriegel M.A., Rathinam C., Flavell R.A. (2009). E3 ubiquitin ligase GRAIL controls primary T cell activation and oral tolerance. Proc. Natl. Acad. Sci. USA.

[193] Johannes L., Wunder C. (2011). Retrograde transport: Two (or more) roads diverged in an endosomal tree?. Traffic.

[194] Jahn R., Scheller R.H. (2006). SNAREs—Engines for membrane fusion. Nat. Rev. Mol. Cell Biol..

[195] Ganley I.G., Espinosa E., Pfeffer S.R. (2008). A syntaxin 10-SNARE complex distinguishes two distinct transport routes from endosomes to the trans-Golgi in human cells. J. Cell Biol..

[196] Mallard F., Tang B.L., Galli T., Tenza D., Saint-Pol A., Yue X., Antony C., Hong W., Goud B., Johannes L. (2002). Early/recycling endosomes-to-TGN transport involves two SNARE complexes and a Rab6 isoform. J. Cell Biol..

[197] Nakamura N., Fukuda H., Kato A., Hirose S. (2005). MARCH-II is a syntaxin-6-binding protein involved in endosomal trafficking. Mol. Biol. Cell.

[198] Fukuda H., Nakamura N., Hirose S. (2006). MARCH-III Is a novel component of endosomes with properties similar to those of MARCH-II. J. Biochem..

[199] Morokuma Y., Nakamura N., Kato A., Notoya M., Yamamoto Y., Sakai Y., Fukuda H., Yamashina S., Hirata Y., Hirose S. (2007). MARCH-XI, a novel transmembrane ubiquitin ligase implicated in ubiquitin-dependent protein sorting in developing spermatids. J. Biol. Chem..

[200] Meyer C., Zizioli D., Lausmann S., Eskelinen E.L., Hamann J., Saftig P., von Figura K., Schu P. (2000). mu1A-adaptin-deficient mice: Lethality, loss of AP-1 binding and rerouting of mannose 6-phosphate receptors. EMBO J..

[201] Ramalho-Santos J., Schatten G., Moreno R.D. (2002). Control of membrane fusion during spermiogenesis and the acrosome reaction. Biol. Reprod..

[202] Moreno R.D. (2003). Differential expression of lysosomal associated membrane protein (LAMP-1) during mammalian spermiogenesis. Mol. Reprod. Dev..

[203] Martinez-Menarguez J.A., Geuze H.J., Ballesta J. (1996). Evidence for a nonlysosomal origin of the acrosome. J. Histochem. Cytochem..

[204] Kang-Decker N., Mantchev G.T., Juneja S.C., McNiven M.A., van Deursen J.M. (2001). Lack of acrosome formation in Hrb-deficient mice. Science.

[205] Yao R., Ito C., Natsume Y., Sugitani Y., Yamanaka H., Kuretake S., Yanagida K., Sato A., Toshimori K., Noda T. (2002). Lack of acrosome formation in mice lacking a Golgi protein, GOPC. Proc. Natl. Acad. Sci. USA.

[206] Scott P.M., Bilodeau P.S., Zhdankina O., Winistorfer S.C., Hauglund M.J., Allaman M.M., Kearney W.R., Robertson A.D., Boman A.L., Piper R.C. (2004). GGA proteins bind ubiquitin to facilitate sorting at the trans-Golgi network. Nat. Cell Biol..

[207] Puertollano R., Bonifacino J.S. (2004). Interactions of GGA3 with the ubiquitin sorting machinery. Nat. Cell Biol..

[208] Berruti G., Ripolone M., Ceriani M. (2010). USP8, a regulator of endosomal sorting, is involved in mouse acrosome biogenesis through interaction with the spermatid ESCRT-0 complex and microtubules. Biol. Reprod..

[209] Haraguchi C.M., Mabuchi T., Hirata S., Shoda T., Hoshi K., Yokota S. (2004). Ubiquitin signals in the developing acrosome during spermatogenesis of rat testis: An immunoelectron microscopic study. J. Histochem. Cytochem..

[210] Westermann B. (2010). Mitochondrial fusion and fission in cell life and death. Nat. Rev. Mol. Cell Biol..

[211] Hales K.G., Fuller M.T. (1997). Developmentally regulated mitochondrial fusion mediated by a conserved, novel, predicted GTPase. Cell.

[212] Smirnova E., Griparic L., Shurland D.L., van der Bliek A.M. (2001). Dynamin-related protein Drp1 is required for mitochondrial division in mammalian cells. Mol. Biol. Cell.

[213] Bleazard W., McCaffery J.M., King E.J., Bale S., Mozdy A., Tieu Q., Nunnari J., Shaw J.M. (1999). The dynamin-related GTPase Dnm1 regulates mitochondrial fission in yeast. Nat. Cell Biol..

[214] Otsuga D., Keegan B.R., Brisch E., Thatcher J.W., Hermann G.J., Bleazard W., Shaw J.M. (1998). The dynamin-related GTPase, Dnm1p, controls mitochondrial morphology in yeast. J. Cell Biol..

[215] Wong E.D., Wagner J.A., Gorsich S.W., McCaffery J.M., Shaw J.M., Nunnari J. (2000). The dynamin-related GTPase, Mgm1p, is an intermembrane space protein required for maintenance of fusion competent mitochondria. J. Cell Biol..

[216] Shepard K.A., Yaffe M.P. (1999). The yeast dynamin-like protein, Mgm1p, functions on the mitochondrial outer membrane to mediate mitochondrial inheritance. J. Cell Biol..

[217] Hermann G.J., Thatcher J.W., Mills J.P., Hales K.G., Fuller M.T., Nunnari J., Shaw J.M. (1998). Mitochondrial fusion in yeast requires the transmembrane GTPase Fzo1p. J. Cell Biol..

[218] Rapaport D., Brunner M., Neupert W., Westermann B. (1998). Fzo1p is a mitochondrial outer membrane protein essential for the biogenesis of functional mitochondria in Saccharomyces cerevisiae. J. Biol. Chem..

[219] Olichon A., Baricault L., Gas N., Guillou E., Valette A., Belenguer P., Lenaers G. (2003). Loss of OPA1 perturbates the mitochondrial inner membrane structure and integrity, leading to cytochrome c release and apoptosis. J. Biol. Chem..

[220] Santel A., Fuller M.T. (2001). Control of mitochondrial morphology by a human mitofusin. J. Cell Sci..

[221] Chen H., Detmer S.A., Ewald A.J., Griffin E.E., Fraser S.E., Chan D.C. (2003). Mitofusins Mfn1 and Mfn2 coordinately regulate mitochondrial fusion and are essential for embryonic development. J. Cell Biol..

[222] Alexander C., Votruba M., Pesch U.E., Thiselton D.L., Mayer S., Moore A., Rodriguez M., Kellner U., Leo-Kottler B., Auburger G., Bhattacharya S.S., Wissinger B. (2000). OPA1, encoding a dynamin-related GTPase, is mutated in autosomal dominant optic atrophy linked to chromosome 3q28. Nat. Genet..

[223] Delettre C., Lenaers G., Griffoin J.M., Gigarel N., Lorenzo C., Belenguer P., Pelloquin L., Grosgeorge J., Turc-Carel C., Perret E. (2000). Nuclear gene OPA1, encoding a mitochondrial dynamin-related protein, is mutated in dominant optic atrophy. Nat. Genet..

[224] Zuchner S., Mersiyanova I.V., Muglia M., Bissar-Tadmouri N., Rochelle J., Dadali E.L., Zappia M., Nelis E., Patitucci A., Senderek J. (2004). Mutations in the mitochondrial GTPase mitofusin 2 cause Charcot-Marie-Tooth neuropathy type 2A. Nat. Genet..

[225] Wakabayashi J., Zhang Z., Wakabayashi N., Tamura Y., Fukaya M., Kensler T.W., Iijima M., Sesaki H. (2009). The dynamin-related GTPase Drp1 is required for embryonic and brain development in mice. J. Cell Biol..

[226] Ishihara N., Nomura M., Jofuku A., Kato H., Suzuki S.O., Masuda K., Otera H., Nakanishi Y., Nonaka I., Goto Y. (2009). Mitochondrial fission factor Drp1 is essential for embryonic development and synapse formation in mice. Nat. Cell Biol..

[227] Rinaldi T., Ricci C., Porro D., Bolotin-Fukuhara M., Frontali L. (1998). A mutation in a novel yeast proteasomal gene, RPN11/MPR1, produces a cell cycle arrest, overreplication of nuclear and mitochondrial DNA, and an altered mitochondrial morphology. Mol. Biol. Cell.

[228] Fisk H.A., Yaffe M.P. (1999). A role for ubiquitination in mitochondrial inheritance in Saccharomyces cerevisiae. J. Cell Biol..

[229] Escobar-Henriques M., Westermann B., Langer T. (2006). Regulation of mitochondrial fusion by the F-box protein Mdm30 involves proteasome-independent turnover of Fzo1. J. Cell Biol..

[230] Fritz S., Weinbach N., Westermann B. (2003). Mdm30 is an F-box protein required for maintenance of fusion-competent mitochondria in yeast. Mol. Biol. Cell.

[231] Cohen M.M., Amiott E.A., Day A.R., Leboucher G.P., Pryce E.N., Glickman M.H., McCaffery J.M., Shaw J.M., Weissman A.M. (2011). Sequential requirements for the GTPase domain of the mitofusin Fzo1 and the ubiquitin ligase SCFMdm30 in mitochondrial outer membrane fusion. J. Cell Sci..

[232] Cohen M.M., Leboucher G.P., Livnat-Levanon N., Glickman M.H., Weissman A.M. (2008). Ubiquitin-proteasome-dependent degradation of a mitofusin, a critical regulator of mitochondrial fusion. Mol. Biol. Cell.

[233] Nakamura N., Kimura Y., Tokuda M., Honda S., Hirose S. (2006). MARCH-V is a novel mitofusin 2- and Drp1-binding protein able to change mitochondrial morphology. EMBO Rep..

[234] Yonashiro R., Ishido S., Kyo S., Fukuda T., Goto E., Matsuki Y., Ohmura-Hoshino M., Sada K., Hotta H., Yamamura H., Inatome R., Yanagi S. (2006). A novel mitochondrial ubiquitin ligase plays a critical role in mitochondrial dynamics. EMBO J..

[235] Karbowski M., Neutzner A., Youle R.J. (2007). The mitochondrial E3 ubiquitin ligase MARCH5 is required for Drp1 dependent mitochondrial division. J. Cell Biol..

[236] Park Y.Y., Lee S., Karbowski M., Neutzner A., Youle R.J., Cho H. (2010). Loss of MARCH5 mitochondrial E3 ubiquitin ligase induces cellular senescence through dynamin-related protein 1 and mitofusin 1. J. Cell Sci..

[237] Tanaka A., Cleland M.M., Xu S., Narendra D.P., Suen D.F., Karbowski M., Youle R.J. (2010). Proteasome and p97 mediate mitophagy and degradation of mitofusins induced by Parkin. J. Cell Biol..

[238] Heo J.M., Livnat-Levanon N., Taylor E.B., Jones K.T., Dephoure N., Ring J., Xie J., Brodsky J.L., Madeo F., Gygi S.P. (2010). A stress-responsive system for mitochondrial protein degradation. Mol. Cell.

[239] Xu S., Peng G., Wang Y., Fang S., Karbowski M. (2011). The AAA-ATPase p97 is essential for outer mitochondrial membrane protein turnover. Mol. Biol. Cell.

[240] Karbowski M., Youle R.J. (2011). Regulating mitochondrial outer membrane proteins by ubiquitination and proteasomal degradation. Curr. Opin. Cell Biol..

[241] Chai Y., Koppenhafer S.L., Shoesmith S.J., Perez M.K., Paulson H.L. (1999). Evidence for proteasome involvement in polyglutamine disease: Localization to nuclear inclusions in SCA3/MJD and suppression of polyglutamine aggregation *in vitro*. Hum. Mol. Genet..

[242] Shaw B.F., Valentine J.S. (2007). How do ALS-associated mutations in superoxide dismutase 1 promote aggregation of the protein?. Trends Biochem. Sci..

[243] Sugiura A., Yonashiro R., Fukuda T., Matsushita N., Nagashima S., Inatome R., Yanagi S. (2011). A mitochondrial ubiquitin ligase MITOL controls cell toxicity of polyglutamine-expanded protein. Mitochondrion.

[244] Yonashiro R., Sugiura A., Miyachi M., Fukuda T., Matsushita N., Inatome R., Ogata Y., Suzuki T., Dohmae N., Yanagi S. (2009). Mitochondrial ubiquitin ligase MITOL ubiquitinates mutant SOD1 and attenuates mutant SOD1-induced reactive oxygen species generation. Mol. Biol. Cell.

[245] Scott I. (2010). The role of mitochondria in the mammalian antiviral defense system. Mitochondrion.

[246] Tal M.C., Iwasaki A. (2011). Mitoxosome: A mitochondrial platform for cross-talk between cellular stress and antiviral signaling. Immunol. Rev..

[247] Shi H.X., Liu X., Wang Q., Tang P.P., Liu X.Y., Shan Y.F., Wang C. (2011). Mitochondrial ubiquitin ligase MARCH5 promotes TLR7 signaling by attenuating TANK action. PLoS Pathog..

[248] Braschi E., Zunino R., McBride H.M. (2009). MAPL is a new mitochondrial SUMO E3 ligase that regulates mitochondrial fission. EMBO Rep..

[249] Neuspiel M., Schauss A.C., Braschi E., Zunino R., Rippstein P., Rachubinski R.A., Andrade-Navarro M.A., McBride H.M. (2008). Cargo-selected transport from the mitochondria to peroxisomes is mediated by vesicular carriers. Curr. Biol..

[250] Braschi E., Goyon V., Zunino R., Mohanty A., Xu L., McBride H.M. (2010). Vps35 mediates vesicle transport between the mitochondria and peroxisomes. Curr. Biol..

[251] Youle R.J., Narendra D.P. (2011). Mechanisms of mitophagy. Nat. Rev. Mol. Cell Biol..

[252] Ziviani E., Tao R.N., Whitworth A.J. (2010). Drosophila parkin requires PINK1 for mitochondrial translocation and ubiquitinates mitofusin. Proc. Natl. Acad. Sci. USA.

[253] Poole A.C., Thomas R.E., Yu S., Vincow E.S., Pallanck L. (2010). The mitochondrial fusion-promoting factor mitofusin is a substrate of the PINK1/parkin pathway. PLoS One.

[254] Gegg M.E., Cooper J.M., Chau K.Y., Rojo M., Schapira A.H., Taanman J.W. (2010). Mitofusin 1 and mitofusin 2 are ubiquitinated in a PINK1/parkin-dependent manner upon induction of mitophagy. Hum. Mol. Genet..

[255] Geisler S., Holmstrom K.M., Skujat D., Fiesel F.C., Rothfuss O.C., Kahle P.J., Springer W. (2010). PINK1/Parkin-mediated mitophagy is dependent on VDAC1 and p62/SQSTM1. Nat. Cell Biol..

[256] Tang F., Wang B., Li N., Wu Y., Jia J., Suo T., Chen Q., Liu Y.J., Tang J. (2011). RNF185, a Novel Mitochondrial Ubiquitin E3 Ligase, Regulates Autophagy through Interaction with BNIP1. PLoS One.

[257] Guais A., Solhonne B., Melaine N., Guellaen G., Bulle F. (2004). Goliath, a ring-H2 mitochondrial protein, regulated by luteinizing hormone/human chorionic gonadotropin in rat leydig cells. Biol. Reprod..

